# Neuroprotective Effects of *Glycyrrhiza glabra* Total Extract and Isolated Compounds

**DOI:** 10.3390/ph17070852

**Published:** 2024-06-28

**Authors:** Ali O. E. Eltahir, Sylvester I. Omoruyi, Tanya N. Augustine, Robert C. Luckay, Ahmed A. Hussein

**Affiliations:** 1 Chemistry Department, Cape Peninsula University of Technology, Symphony Rd. Bellville, Cape Town 7535, South Africa; aliomers250@gmail.com; 2 School of Anatomical Sciences, Faculty of Health Sciences, University of the Witwatersrand, Parktown, Johannesburg 2193, South Africa; sylvester.omoruyi@wits.ac.za (S.I.O.); tanya.augustine@wits.ac.za (T.N.A.); 3 Department of Chemistry and Polymer Science, Stellenbosch University, Matieland, Stellenbosch 7602, South Africa; rcluckay@sun.ac.za

**Keywords:** *Glycyrrhiza glabra*, licorice, phenolic compounds, neuroprotection

## Abstract

*Glycyrrhiza glabra* L. is a plant commonly utilized in herbal medicine and stands out as one of the more extensively researched medicinal plants globally. It has been documented with respect to several pharmacological activities, notably, neuroprotective effects, among others. However, the neuroprotective activity of pure phenolic compounds has not been reported yet. The chromatographic of a methanolic extract yielded twenty-two compounds, viz.: naringenin 4′-*O*-glucoside (**1**), 3′,4′,7-trihydroxyflavanone (butin) (**2**), liquiritin (**3**), liquiritin apioside (**4**), abyssinone (**5**), glabrol (**6**), isoliquiritin (**7**), neoisoliquiritin (**8**), isoliquiritin apioside (**9**), licuraside (**10**). 3’[*O*], 4’-(2,2-dimethylpyrano)-3,7-dihydroxyflavanone (**11**), glabrocoumarin (**12**), glabrene (**13**), isomedicarpin (**14**), 7-hydroxy-4′-methoxyflavone (formononetin) (**15**), ononin (**16**), glycyroside (**17**), (3*S*)-7,4′-dihydroxy-2′-methoxyisoflavan (**18**), glabridin (**19**), neoliquiritin (**20**), 3,11-dioxooleana-1,12-dien-29-oic acid (**21**), and 3-oxo-18β-glycyrrhetinic acid (**22**). The results of the neuroprotection evaluation showed that *G. glabra* total extract (TE) and compounds **1**, **7**, **11**, **16**, and **20** protected SH-SY5Y cells by inhibiting the depletion of ATP and elevated caspase 3/7 activities induced by MPP+. Indeed, this study reports for the first time the structure and activity of compound **11** and the neuroprotective activity of some phenolic constituents from *G. glabra*.

## 1. Introduction

Neurodegenerative diseases are a group of diseases characterized by the loss of neurons in the brain [1]. Notable amongst them are Parkinson’s disease and Alzheimer’s disease. Specifically, Parkinson’s disease (PD) involves the loss of dopaminergic neurons in the substantia nigra pars compacta of the midbrain [2]. Classical symptoms of PD include tremors, bradykinesia, akinesia, and postural imbalance [3]. At the molecular level, the progressive loss of dopaminergic neurons is triggered by a cascade of events, including the alteration of the mitochondrial electron transport chain following the accumulation of reactive oxygen species and the loss of adenosine triphosphate (ATP) in the cells [4]. This cascade of events will eventually lead to the death of neurons [5]. Although the actual cause of PD is yet to be fully understood, the onset is believed to result from an interplay between the environment and genetic factors [2]. For instance, exposure to certain neurotoxins such as 1-methyl-4-phenyl-1,2,3,6-tetrahydropyridine (MPTP), a heroin analogue, has been implicated in the onset of the disease [6]. Age is also a major contributing factor, as PD mainly affects people aged 60 and above [7]. To date, treatment of PD remains daunting, as existing treatments only treat symptoms, and levodopa in particular, a dopamine replacement agent which is the current standard care, can nevertheless also lead to Parkinsonism symptoms after prolonged usage [8]. In this regard, medicinal and natural products have continued to serve as a resource base for exploiting biochemical entities that could offer neuroprotective activities [9,10,11].

Licorice is a name given to different species of the *Glycyrrhiza* (Licorice) genus. Notable amongst them is *G. glabra,* one of the most valuable medicinal plants. It consists of about 30 taxa, of which only 15 taxa have been studied so far [12], mainly including the species *G. glabra* L., *G. uralensis* Fisch., *G. inflata* Bat., *G. echinata* L., *G. lepidota.*, *G. pallidflora* Maxim., and *G. macedonica*, [13,14,15]. The plants, renowned for their medicinal properties, are widely used in herbal medicine and are among the most extensively studied medicinal plants globally. They have a notable pharmaceutical history in regions such as China, India, Iran, Spain, Italy, Russia, and North Africa, and remain crucial in the exploration of new pharmaceuticals. Several modern medical research studies have concentrated on their chemical constituents and biological effects, aiming to understand the underlying mechanisms. Numerous pharmacological studies, including clinical investigations, have explored the effects of licorice root extract or its isolated compounds on the nervous system. These plants exhibit various pharmacological activities, including neuroprotective, anti-inflammatory, antimicrobial, anticancer, gastroprotective, hepatoprotective, and cardioprotective effects. Additionally, they have been found to be effective treatments for influenza, cough, lung diseases, pneumonia, bronchitis, skin diseases, and hormone replacement therapy [16,17,18,19,20,21,22,23]. Recently, the plant was used for the treatment of immunostimulating effects in the contexts of coronavirus disease 2019 (COVID-19) and severe acute respiratory syndrome coronavirus 2 (SARS-CoV-2) virus [24,25]. *G. glabra* and *G. uralensis* are the most studied species of this genus. Furthermore, the literature has reported up to 400 compounds from the *Glycyrrhiza* genus, which have been categorized into various classes such as phenolics (flavanones, flavones, flavanonols, chalcones and dihydrochalcones, isoflavones, and coumestans/phenyldihydrocoumarins, and their methoxylated, prenylated, and glycosolated derivatives) and triterpenoids, including their glycosides [26].

Although licorice species have been extensively studied for their biological activities, including their neuroprotective activities, the present study investigates the neuroprotective activities of the TE of *G. glabra* and its isolated compounds in an in vitro model of PD in the neuroblastoma cell line, SH-SY5Y, using the neurotoxin 1-methyl-4-phenylpyridinium (MPP^+^). Indeed, several studies have demonstrated that MPP+, which is a byproduct of MPTP, a toxin known to induce parkinsonism, induces neurotoxicity in the SH-SY5Y cells [27,28]. We also report for the first time the activities of novel compounds isolated and identified from licorice species and show for the first time the neuroprotective activities of these compounds in a PD model.

## 2. Results

### 2.1. Chemical Characterization of the Isolated Compounds

**Figure 1:**
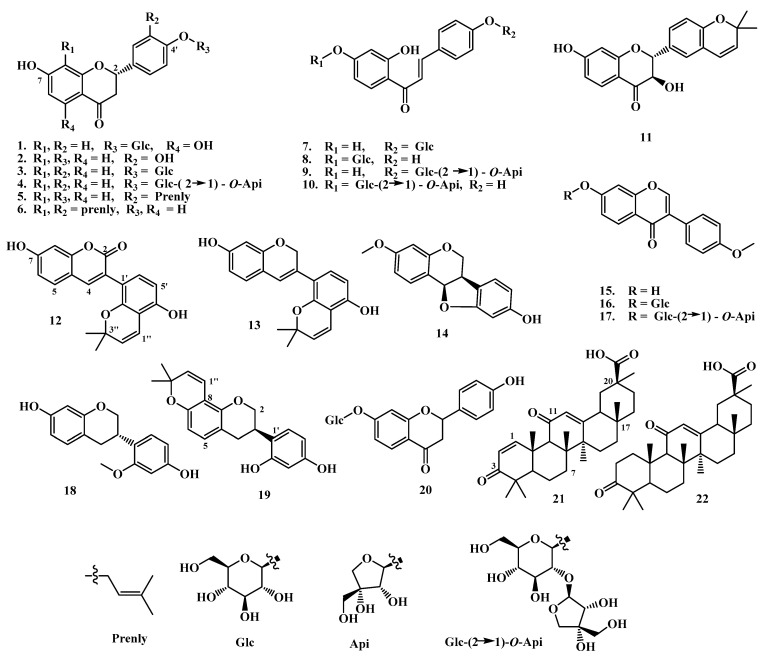
Chemical structures of the compounds isolated from *G. glabra*.

Chromatographic purification of the total extract using different techniques, including semi-prep HPLC, resulted in the isolation of twenty-two (22) pure compounds, which were identified as naringenin 4′-*O*-glucoside (**1**) [29,30], 3′, 4′, 7-trihydroxyflavanone (butin) (**2**) [31,32], liquiritin (**3**) [33,34], liquiritin apioside (**4**) [33], abyssinone (**5**) [35], glabrol (**6**) [33,36,37], isoliquiritin (**7**) [38,39], neoisoliquiritin (**8**) [38,39], isoliquiritin apioside (**9**) [40,41,42], licuraside (**10**) [40,41,42,43], 3’[*O*],4’-(2,2-dimethylpyrano)-3,7-dihydroxyflavanone (**11**), glabrocoumarin (**12**) [44], glabrene (**13**) [37,45], isomedicarpin (**14**) [33,46], 7-hydroxy-4′-methoxyflavone (formononetin) (**15**) [33], ononin (**16**) [30,33], glycyroside (**17**) [30,33], (3*S*)-7,4′-dihydroxy-2′-methoxyisoflavan (**18**) [47], glabridin (**19**) [33,34,37], neoliquiritin (**20**) [48], 3,11-dioxooleana-1,12-die-29-oic acid (**21**) [49], and 3-oxo-18β-glycyrrhetinic acid (22) [50]. The isolated compounds were identified based on detailed spectroscopic analyses and comparison with those reported in the literature (Figure 1).

Compound **11** was isolated as a whitish-yellow powder, and its molecular formula was determined to be C_20_H_18_O_5_ by its HRESIMS (High Resolution Electrospray Ionization Mass Spectrometry). The UV spectrum of **11** had absorption maxima at 313 and 276 nm, whereas the IR spectrum showed absorption bands at 3374, 1673, 1608, 1502, and 1463 cm^−1^ for OH, CO, and aromatic groups. 

**Figure 2:**
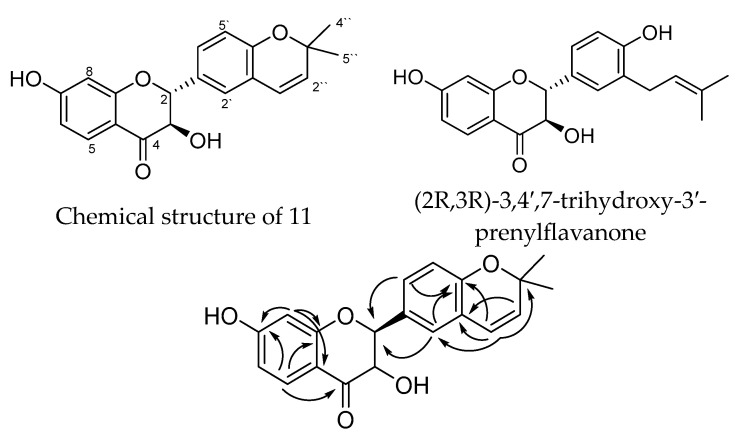
Chemical structure and important HMBC correlations of **11**, and the structure of 3,4′,7-trihydroxy-3′-prenylflavanone.

The ^1^H NMR spectrum of **11** showed signals assignable to six aromatic protons belonging to two 1,3,4-trisubstituted aromatic rings [δ_H_ 7.72 (*d, J* = 8.5 Hz), 6.51 (*dd*, *J* = 8.5, 2.0 Hz), and 6.30 (*d*, *J* = 2.0 Hz); δ_H_ 7.25 (*d*, *J* = 7.9 Hz), and 6.76 (*br d, J* = 7.9 Hz), 7.23 (*br s*)]; two oxygenated methines [δ_H_ 4.92 and 4.50 (*d* each, *J* = 11.8 Hz)], and a 2,2-dimethyl pyrane ring [δ_H_ 6.43 and 5.58 (*d* each, *J* = 9.7 Hz), and two methyls at 1.37 (6H, s)]. The ^13^C NMR and DEPT-135 showed 20 carbons which could be classified as CO (192.7); 14 olefinic signals, 12 of them aromatics (see Table 1); three oxy carbons (83.7, 76.3 and 72.9); and two methyls (27.7, 2C’s). The above data showed similarity with (2*R*,3*R*)-3,4′,7-trihydroxy-3′-prenylflavanone isolated from the same source by Kuroda et. al. (2010) [51]; the only differences are lying on the chemical shifts of the prenyl signals. This similarity indicates that **11** could be a 3-hydroxyflavanol derivative attached to a pyrane ring. Heteronuclear Multiple Bond Correlation (HMBC) correlations between H-5 and C-4, C-7, C-9; H-8/C-7, C-9, C-10; H-6′/C-2, C-4′; H-2′/C-2, C-4′, H-1″/C-4′, C-2′, C-3″ indicate connectivity of the molecule and the linkage of the pyrane ring at C3′, C4′ of ring B (Figure 2). 

The known compounds were identified based on the extensive NMR data, including 2D (HSQC, COSY, and HMBC). The ^1^H NMR spectrum of **1** displayed a typical flavanone skeleton, as well as a 2-3 dihydro ring C pattern [δ_H_ 5.50 (*d*, *J* = 12.7, 2.2 Hz, H-2; δ_C_ 78.4), δ_H_ 2.69 (*dd*, *J* = 17.1, 2.2 Hz, H-3β), and δ_H_ 3.24 (H-3α, overlapped; δ_C_ 42.4)]. The peaks at δ_H_ 7.43 (*d*, *J* = 8.6 Hz, H-2′, 6′) and δ_H_ 7.07 (*d*, *J* = 8.6 Hz, H-3′, 5′) indicated a 1,4-disubstituted ring B. The two protons at δ_H_ 5.85 and 5.83 (*br s*, δ_C_ 95.8 and 96.7) suggested free 6,8-positions of ring A. Additionally, the proton at δ_H_ 4.89 (*d*, *J* = 7.2 Hz, δ_C_ 100.7) was identified as an anomeric proton of a glucose unit. The ^13^C NMR and DEPT-135 spectra revealed 19 signals corresponding to 21 carbons, including a signal at δ_C_ 196.1 (CO, C-4), supporting the presence of a flavanone skeleton. The attachment of the glucose unit to C-4′ is confirmed by the HMBC correlation of the anomeric proton with C-4′. The NMR data (Table S1) are identical to those of naringenin-4′-*O*-glucoside, previously isolated from the same source and *Salvia patens* [29,30].

Compounds **2**−**6** and **20** share a similar flavanone skeleton pattern with compound **1**. Compound **2** has characteristics similar to **1**, with the main differences being the absence of a glucose unit and the splitting of ring B into a typical 1,3,4-trisubstituted benzene ring, identified as 3′,4′,7-trihydroxyflavanone. The compound has been isolated previously from *Vernonia anthelmintica* and *Cotinus coggygria* [31,32]. Compounds **3** and **4** are also flavanones and are connected to a sugar at position C-4′. Compound **3** contains a glucose unit, which is supported by the presence of HMBC correlation between the anomeric proton (δ_H_ 4.89/δc 100.3) and C-4’, while compound **4** exhibits an additional signal of apiose at δ_H_ 5.36/δc 108.7. Both compounds were isolated from the same source [33,34].

Compounds **5** and **6** both exhibit additional signals from prenyl groups. In compound **5**, the prenyl signals are observed at the following chemical shifts (δ_H_/δ_C_): 3.22 (6.5, 2H-1″)/27.9; 5.28 *(br d,* 6.5, H-2″)/123.1; 1.68 (s, Me-4″)/26.0, and 1.67 (s, Me-5″)/18.1, in addition to a signal observed at δ_C_ 131.7 (C-3″). The prenyl group is attached to C-3′, as confirmed by HMBC correlation. Compound **6**, on the other hand, features two prenyl groups, at positions C-3′ and -8. (see Table S1 for more details). Both compounds **5** [35] and **6** [33,36,37] were derived from the same source. Compound **20** shares a similar profile with compound **3**, the only distinction being the attachment of the glucose unit at C-7 instead of C-3′ (as in compound **3**) [48].

Compounds **7**–**10** exhibit the same NMR pattern and are closely related to previous compounds, particularly compounds **2**, **3**, and **20**. However, in these compounds, ring C is replaced by a 1,4-enone system, as indicated by the signals corresponding to the carbonyl (CO), C_α_ and C_β_. The change of glucose link between C-4 and C-4′ is evident and supported by HMBC correlations between the anomeric protons and the corresponding carbons [42,43]. For compounds **9** and **10**, the situation is analogous, with the glucose moiety being replaced by a glucose–apiose moiety. This replacement is supported by the similar NMR patterns and HMBC correlations, providing clear evidence of the structural changes [40,41,42].

Compound **12** exhibited a 1,2-benzopyrone nucleus (coumarin) with an OH group at C-7. The signal at δ_H_ 7.82/δ_C_ 142.5 indicates H-4, while the *o*-coupled protons at δ_H_ 7.52 (δ_C_ 129.6) and δ_H_ 6.79 (δ_C_ 113.1) correspond to H-5 and H-6. The *o*-coupled protons at δ_H_ 6.32 (δ_C_ 107.3) and δ_H_ 6.94 (δ_C_ 130.6) indicate H-5′ and H-6′, belonging to a 1,2,3,4-tetrasubstituted ring B. Additionally, the signals at δ_H_ 6.57 (d, *J* = 10 Hz, H-1″, δ_C_ 116.8), δ_H_ 5.53 (*d*, *J* = 10 Hz, H-2″, δ_C_ 128.3), and the two methyl groups at δ_H_ 1.37 (6H, s, δ_C_ 27.2 × 2C) indicate a pyrane ring attached to C-3 and C-2 of ring B. The data (see Table S1) are consistent with glabrocoumarins, and confirmed by 2D spectra. The compound has been previously isolated from the same source [44].

Compound **13** displayed signals similar to **12**, with the only difference being the absence of the carbonyl group at C-2 and the formation of another 2*H*-pyran ring. The data (see Table S1) are consistent with glabrene, also previously isolated from the same source [37,45].

Compound **14** displayed signals corresponding to a 1,2,4-trisubstituted benzene ring (see Table S1), as well as a 3,4-dihydro-2*H*-pyran ring [δ_H_ 3.56, 4.16 (CH_2_-2)/δ_C_ 68.5; δ_H_ 3.52 (H-3)/δ_C_ 39.3; δ_H_ 5.47 (H-4)/δ_C_ 78.0]. Additionally, it showed an OMe group, at δ_H_ 3.65/δ_C_ 55.2, connected to C-7. These findings are consistent with isomedicarpin, which was isolated from the same source [33,46].

Compounds **15**–**17** share the same isoflavonoid skeleton, as indicated by the H-2 signals (δ_H_ ~ 8.40). Ring A is connected to different side chains at C-7, while ring B is 1,4-disubstituted with a methoxy group at C-4′ (see Table S1 for more details). Compound **15** has a hydroxyl group at C-7, compound **16** has a glucose moiety, and compound **17** has a glucose–apiose moiety, as evidenced by the anomeric H/C signals of the sugars. These three compounds were previously isolated from the same source [30,33].

Compound **18** displayed two 1,2,4-trisubstituted benzene rings (rings A and B) and a tetrahydropyrane ring (ring C), as evidenced by the presence of signals for two methylenes (δ_H_ 4.14, 4.61; δ_C_ 69.2 and δ_H_ 2.71, 2.87; δ_C_ 29.7) and a methine (δ_H_ 3.32; δ_C_ 31.0). Ring A showed signals for two *o*-coupled protons (δ_H_ 6.85, d, *J* = 8.4 Hz, H-5 and δ_H_ 6.29, br *d*, *J* = 8.4 Hz, H-6) and a singlet at δ_H_ 6.19 (*br s*, H-8). Ring B exhibited signals for two *o*-coupled protons (δ_H_ 6.34, *br d*, *J* = 8.6 Hz, H-5′ and δ_H_ 6.97, *d*, *J* = 8.6 Hz, H-6′) and a singlet at δ_H_ 6.43 (*br s*, H-3′). The ^13^C NMR and DEPT-135 spectra showed 16 well-resolved signals, corresponding to 16 carbons, including two methylenes, one *sp*^3^ methine, six *sp*^2^ methines, six *sp^2^* quaternary carbons, and a methoxy group. The NMR data matched those of 7,4′-dihydroxy-2′-methoxyisoflavan isolated from propolis [47]. Compound **19** is similar to compound **18**, with the only difference being the absence of the methoxy group at C-2′ and the appearance of a pyrane ring at C-8 and C-7 of ring A. The NMR data for compound **19** matched those of glabridin, which was isolated from the same source (see Table S1 for more details) [33,34,37].

Compounds **21** and **22** are oleanane-type triterpenes. Compound **22** exhibited seven methyl singlet signals at δ_H_/δ_C_: 1.12/26.3 (C-23), 1.09/21.4 (C-24), 1.29/15.6 (C-25), 1.19/18.5 (C-26), 1.40/23.3 (C-27), 0.87/28.5 (C-28), and 1.25/28.4 (C-29), along with a singlet at 5.47 (H-12). The ^13^C NMR spectrum showed 29 well-resolved signals corresponding to 30 carbons, including two carbonyl carbons, at 217.0 and 199.7 ppm, the latter conjugated with a double bond at 169.8/128.4 ppm. Additionally, the spectrum included three methines, nine methylenes, and five quaternary carbons. The presence of the carbonyl carbon of the carboxylic group at 181.1 ppm and the conjugated double bond are characteristic fragments of glycyrrhetinic acid isolated from the same source. Based on the NMR data, the compound was identified as 3-oxo-glycyrrhetinic acid [50], with data matching reported values. It is important to note that, based on 2D NMR correlations, some chemical shifts were reassigned and corrected from reference [50], particularly for C-6, C-7, C-8, and C-14, and methyl groups at C-28 and C-29.

The NMR data for compound **21** are identical to those of compound **22**, with the exception of the presence of a double bond in ring A. This double bond is supported by signals at δ_H_/δ_C_ 7.72/161.6 (both doublets, *J* = 10.4 Hz), and the high chemical shift of C-3 (204.6 ppm), as compared to compound **22**. Through the analysis of various 2D NMR experiments, a complete assignment of the carbons and protons was achieved (see Table S1). Compound **21** was identified as 3,11-dioxooleana-1,12-die-29-oic acid, which has been reported previously as a synthetic derivative [49]. Notably, this is the first comprehensive report of the NMR data for compound **21**.

The purity of the isolated compounds was confirmed by NMR, TLC, and HPLC (purity ≥ 95% for all compounds). Compound **11** is reported for the first time in this study, while **21** was reported previously as a synthetic product [49].

### 2.2. Biological Study

#### 2.2.1. Dose–Response of Licorice TE and Compounds

**Figure 3:**
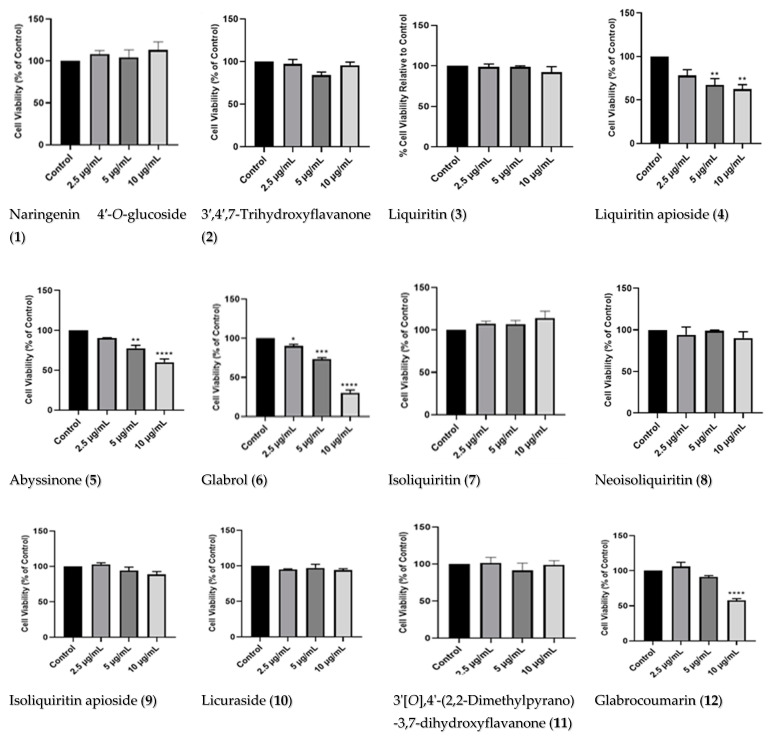

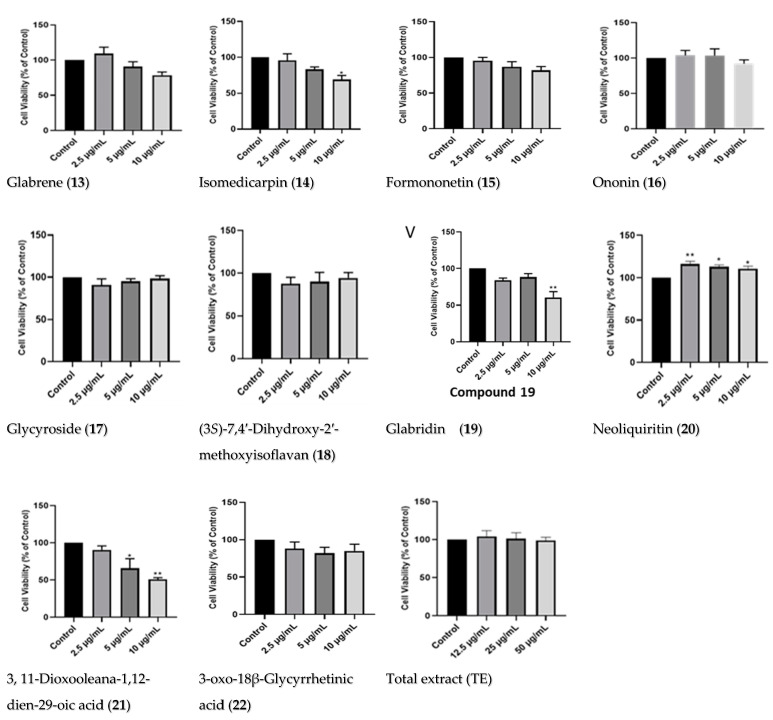
Dose–response of licorice TE and compounds. MTT cytotoxicity assay on SH-SY5Y cells treated with increasing concentrations (12.5, 25, and 50 µg/mL) of licorice TE and compounds (2.5, 5, and 10 µg/mL) for 24 h. After assays, cell viability was expressed as a percentage of control, and each bar represents the mean + SEM of at least three replicate experiments obtained from quadruple wells. Treated cells were compared to control cells; significance levels are indicated. The significance of the difference when control cells were compared to treated cells is indicated by * *p*< 0.05, ** *p* < 0.01, *** *p* < 0.001, and **** *p* < 0.0001.

To investigate the neuroprotective potentials of the TE and compounds, their effects on the cell viability of the SH-SY5Y were first determined. The total extract and 22 compounds were screened for their cytotoxicity using concentrations of 12.5, 25, and 50 μg/mL for the extract, and 2.5, 5, and 10 μg/mL for the compounds. The results show that the TE and compounds had minimal effects on the cell viability of the SH-SY5Y cells, especially at 2.5 μg/mL; however, with increased concentration, some compounds induced toxicity (Figure 3). In addition, the cells treated with compound 20 showed increased cell viability for all concentrations. In contrast, the cells treated with compound 6 showed significant reductions in cell viability at all concentrations tested. Following these results, the TE and five compounds, **1**, **7**, **11**, **16** and **20**, were selected to be investigated for their neuroprotective potentials, as they were not cytotoxic, and they improved cell viability the most compared to other compounds. In particular, compound **20** significantly increased cell viability at all concentrations tested.

#### 2.2.2. Effects of Licorice TE and Compounds on MPP+-Induced Toxicity on SH-SY5Y Cells

**Figure 4:**
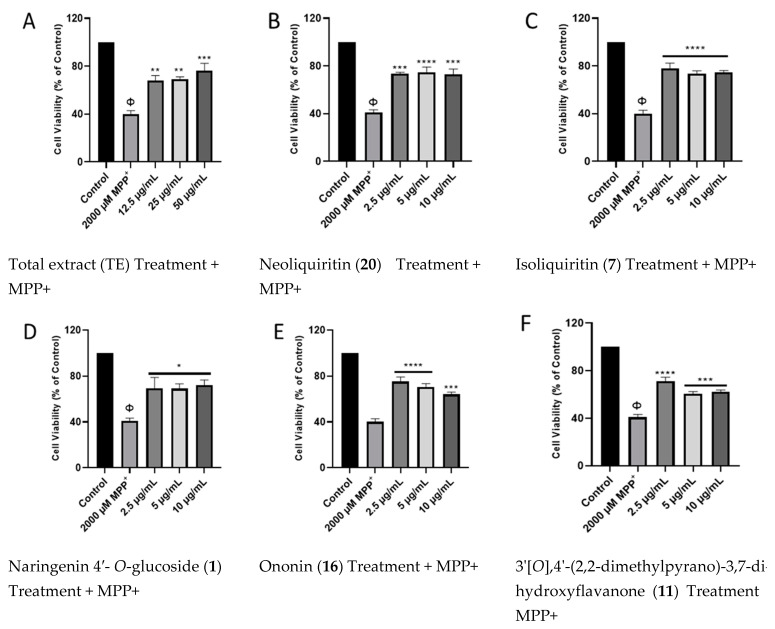
Licorice TE and compounds show protection in SH-SY5Y cells. Cells were pre-treated with TE (**A**) and compounds (**B**–**F**) before exposure to MPP^+^ for 24 h. Each bar represents mean percentage cell viability relative to control, and significance of difference is indicated with * *p* < 0.05, ** *p* < 0.01, *** *p* < 0.001, and **** *p* < 0.0001 when TE/compounds are compared to MPP+, and Φ when MPP^+^ -treated cells are compared to control.

To investigate the neuroprotective activities of the TE and compounds, their effects on cell viability in the presence of the MPP^+^ neurotoxin were determined using the MTT cell viability assay. The results show that pretreatment with the TE and compounds resulted in significant improvements in cell viability of MPP+-treated cells, compared to cells treated with MPP alone (Figure 4). While MPP+ reduced cell viability to about 40% when compared to the control cells, pretreatment with the TE increased cell viability to 63, 69, and 73% for 12.5, 25, and 50 μg/mL, respectively (Figure 4A). Although all tested concentrations showed improvement in cell viability, the increase was more in 2.5 μg/mL concentrations for most of the compounds, and in 50 μg/mL for the TE. Considering this, the concentrations of 50 and 2.5 μg/mL were chosen for the TE and compounds, respectively, for further studies. Together, these results show that licorice TE and compounds confer neuroprotection on the SH-SY5Y cells.

#### 2.2.3. Effect of TE and Compounds on ATP Production in the Cells

**Figure 5:**
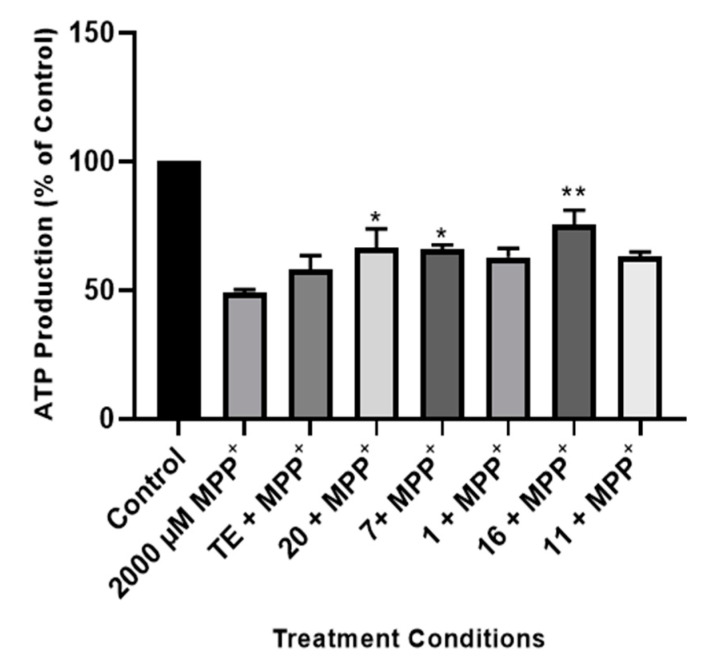
Effect of licorice TE and compounds on MPP+-induced depletion of ATP. Cells were pre-treated with 50 µg/mL of licorice TE and 2.5 µg/mL of compounds before exposure to 2000 µM of MPP+ for 24 h, and ATP levels were assessed. Each bar represents the mean percentage of ATP production relative to control, and the significance of the difference is indicated as * *p* < 0.05 and ** *p* < 0.01 when extract/compounds are compared to MPP+ and when MPP+-treated cells are compared to control.

As a mechanism of neuronal toxicity, MPP+ induces ATP degeneration in neuronal cells by the inhibition of mitochondrial complex I [52]. To understand the mechanism of action of the licorice TE and compounds **1**, **7**, **11**, **16**, and **20**, we next investigated their effects on the levels of ATP in the cells following treatment with the MPP+ neurotoxin. Cells were plated in white-walled 96-well plates and treated with 50 μg/mL of the TE and 2.5 μg/mL of the compounds, as these concentrations showed better neuroprotective activity; ATP production was then measured using the MitotoxGlo Promega ATP kit. Figure 5 shows that MPP^+^ treatment reduced ATP production in the cells to about 49%. However, pretreatment with the licorice TE and compounds improved ATP production in the cells undergoing MPP^+^ treatment, while this was only significant for the cells pretreated with compounds **20**, **7** and **16**, with ATP levels of about 66, 65, and 75%, respectively. These results suggest that improving cellular ATP production is part of the mechanism of neuroprotection conferred by the extract and compounds.

#### 2.2.4. Effect of TE and Compounds on Caspase 3/7 Activities in the Cells

**Figure 6:**
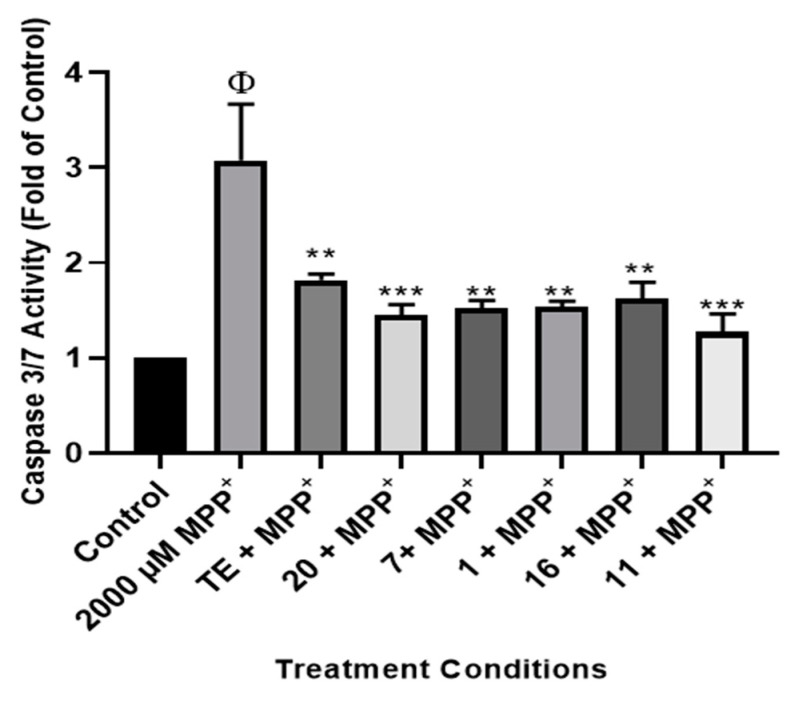
Licorice TE and compounds attenuate MPP+-induced increase in caspase 3/7 activities. Cells were pre-treated with 50 µg/mL of licorice TE and 2.5 µg/mL of compounds before exposure to 2000 µM of MPP+ for 24 h, and caspase 3/7 activities were determined. Each bar represents the level of caspase 3/7 expressed as a multiple of the control, and significance of difference is indicated by ** *p* < 0.01 and *** *p* < 0.001 when TE/compounds were compared to MPP+, and Φ when MPP+-treated cells were compared to control.

To further ascertain the mechanism involved in the neuroprotective aspects of licorice TE and compounds in SH-SY5Y cells, the levels of apoptosis were assessed using caspase 3/7 as a marker. Caspases belong to the family of cysteine proteases, which drive apoptosis in cells and carry out their function by cleavage of substrates; thus, they are used as markers of apoptosis [24,53]. To investigate apoptosis, cells were pretreated with 50 µg/mL of the TE and 2.5 µg/mL of the compounds before exposure to MPP+, and caspase 3/7 activities were measured. Figure 6 shows that TE and compounds mitigate MPP+-increased levels of caspase 3/7 activity in the SH-SY5Y cells. Specifically, MPP+ increased levels of caspase 3/7 to about 3-fold of control and pre-treatment with TE reduced this activity to about 1.8-fold of control. Furthermore, all compounds also protected SH-Y5Y cells from MPP+-induced increase in caspase 3/7 activities. Altogether, these results indicate that inhibition of apoptosis is involved in the neuroprotective aspects of licorice TE and compounds.

## 3. Discussion

The burden of PD continues to pose a challenge to the ageing population globally [54]. While the exact cause of the disease has yet to be fully elucidated, it has been proposed that PD is caused by a plethora of environmental events and genetic factors [55]. In clinical settings, PD is incurable, as it involves different clinical manifestations, including bradykinesia, resting tremor, and rigidity, along with postural instability; hence, medications are given to relieve symptoms [56]. One notable example of such medication is levodopa, but its prolonged use is associated with neuronal cell toxicity and other serious side effects, such as dyskinesias and psychosis [54]. Delaying the onset of PD is thought to effectively slow down the progression of the disease, so exploiting new approaches to achieve this remains critical. 

In the present study, the neuroprotective effects of licorice TE and compounds were evaluated in an MPP^+^ model of PD. Twenty-two compounds were isolated from the plant, with compound **11** being a new compound. The compounds were screened for their cytotoxicity, and some of them were observed to be cytotoxic to the SH-SY5Y cells. These compounds include glabrol (**6**), abyssinone (**5**) [35], liquiritin apioside (**4**), glabrocoumarin (**12**), isomedicarpin (**14**), and 3,11-dioxooleana-1,12-die-29-oic acid (**21**). In support of these findings, liquiritin apioside has been reported to induce toxicity on human cancer cell lines (HSC-2, HSC-3, HSC-4, and HL-60) which are derived from oral squamous cancers of the tongue [57], while glabrol showed cytotoxicity against C6 rat glioma cells [58]. In addition, 18β-glycyrrhetinic acid and formononetin have been reported to have cytotoxic potential against various cancer cell lines [59,60,61,62,63,64]. Despite the cytotoxicity observed for the compounds mentioned above, other compounds from the *Glycyrrhiza* genus have been demonstrated to have neuroprotective effects [20,21,22,23]. To this end, our findings show that the compounds naringenin 4′-*O*-glucoside (**1**), neoliquiritin (**20**), glabrol (**6**), isoliquiritin (**7**), and 3′[*O*],4′-(2,2-dimethylpyrano)-3,7-dihydroxyflavanone (**11**), alongside the TE, when investigated for their neuroprotective potentials, conferred neuroprotection on the cells. Supporting our findings, studies have demonstrated that neoliquiritin (**20**) protected rat cardiac myocytes from doxorubicin-induced reduction in cell proliferation [65], while naringenin, which is the parent compound from which neoliquiritin (**20**) and naringenin 4′-*O*-glucoside (**1**) were derived, is widely known to have cytoprotective potentials, including protecting neurons from toxin-mediated cell death in Alzheimer’s disease and PD [66,67,68]. Isoliquiritin, on the other hand, has also shown to have neuroprotective potentials, as it inhibits monoamine oxidase and ameliorates depression [69,70], while 7-hydroxy-4′-methoxyflavone (formononetin) (**15**) was able to alleviate neuroinflammation in lipopolysaccharide-stimulated microglial cells by inhibiting TLR4/MyD88/MAPK signaling and activating the Nrf2/NQO-1 pathway [71].

As a mechanism of action, MPP+ is known to alter cellular energy levels in neuronal cells following the induction of oxidative stress [72]. Oxidative stress leads to impairment of the mitochondrial electron transport chain and, consequently, a decline in ATP production, as well as increased mitochondrial dysfunction [73,74]. Alteration of cellular ATP negatively impacts the ability of cells to carry out their normal metabolic functions, and in relation to humans, this could lead to cognitive decline, as neurotransmission will be affected [75]. Importantly, studies have shown that the ability to ameliorate oxidative stress and improve cellular energy and metabolism counteracts the effects of neurotoxins and, at the same time, improves neurotransmission and neuron function [75,76]. In line with this postulate, we show that the tested compounds and TE improved cellular ATP levels following exposure to MPP+. In support of these findings, it has been suggested that isoliquiritin provides protective action against corticosterone-induced cell damage by reducing oxidative stress and regulating mitochondrial dysfunction by preventing dissipation of mitochondrial membrane potential [77].

Consequently, neuronal cells proceed to programmed cell death following a decline in ATP, and this contributes to the reduction of dopaminergic neuronal cells in the substantia nigra pars compacta of the midbrain [72,78]. Thus, slowing or preventing neurons with respect to cell death via several mechanisms holds a future in the management of neurodegenerative diseases like PD. In the present study, our findings show that *G. glabra* TE and compounds **20**, **7**, **1**, **16**, and **11** inhibited elevated caspase 3/7 activities induced by MPP+. While this was the case for our study, previous studies have demonstrated that prunin (**20**) exerts its cytoprotective potential by inhibiting apoptosis triggered by cellular toxins in cardiac cells [65], In addition, naringenin showed hepatoprotective and neuroprotective properties against lead-induced oxidative stress, inflammation, and apoptosis in rats [68]. Moreover, isoliquiritin was also demonstrated to attenuate apoptosis by the inhibition of the overload of intracellular calcium Ca^2+^ ions; down-regulation of Bax, caspase 3, and cytochrome protein expression; and up-regulation of Bcl protein expression [77]. Thus, the findings are sufficient to state that prevention of cellular apoptosis is a critical component of the neuroprotective mechanism of *G. glabra* and compounds.

## 4. Materials and Methods

### 4.1. Chemicals, Materials, and Reagents 

Root powder of *G. glabra* was purchased from the local market in Khartoum, Sudan in May 2020; the specimen (H614) was identified by Prof Hatil Elkamali (Department of Botany, Omdurman Islamic University, Omdurman, Sudan). Organic solvents, including methanol, acetonitrile (HPLC grade, Merck, Cape Town, South Africa), hexane, dichloromethane, ethanol, and ethyl acetate (analytical grade (AR), obtained from a local merchant (Kimix, Cape Town, South Africa)) were acquired. Silica gel 60 (0.063–0.200 mm), Sephadex (LH-20), and Aluminum TLC plate, silica gel PF254 were supplied by Merck (Cape Town, South Africa). The 1D NMR (^1^H, ^13^C and DEPT-135) and 2D spectra were measured using a Bruker spectrometer (Rheinstetten, Germany) operating at 400 MHz (for ^1^H) and 100 MHz (for ^13^C). 

### 4.2. Method

#### Extraction and Purification of Compounds 

The root powder (0.5 Kg) was extracted with methanol at 60 °C (3 L × 2 h × 2 time). After concentration, it yielded ~104.3 g. A quantity of 50.0 g of the total extract (TE) was applied to the silica gel column and eluted using a gradient of hexane and ethyl acetate with increasing polarity. Collected fractions were pooled together according to their profiles on the TLC to afford 24 major fractions coded as I to XXIV. The fractions were re-chromatographed to yield twenty-three major compounds as follows: Fraction XXIII (3.50 g) underwent chromatography on silica gel using a hexane: EtOAc gradient (80:20 to 0:100). Subsequently, sub-fraction 10 was subjected to purification on Sephadex with an isocratic 80% aqueous ethanol, followed by semi-preparative HPLC (Shimadzu, Kyoto, Japan) utilizing a MeOH and de-ionized water (DIW) gradient (40:60 to 60:80 in 30 min, then to 80:90 in 10 min, and finally to 100% MeOH in 10 min). This process yielded compounds **1** (4.8 mg), **3** (100.8 mg), **7** (45.6 mg), **8** (80.3 mg), and **16** (5.2 mg).

The main fraction, XXIV (4.30 g), was chromatographed on silica gel, and subsequent subfractions 2 and 3 were individually subjected to chromatography on Sephadex and semi-prep HPLC, as described earlier. This resulted in the isolation of compounds **4** (50.3 mg), **9** (39.8 mg), **10** (20 mg), **17** (15.6 mg), and **20** (5.3 mg).

Fraction XV (2.40 g) underwent chromatography on silica gel. Subfraction 16 was subsequently chromatographed on Sephadex and subjected to semi-prep HPLC as outlined before, resulting in the isolation of compound **2** (10.5 mg).

Fraction X (1.40 g) was chromatographed on silica gel using a hexane:EtOAc gradient (80:20 to 0:100), and sub-fraction 7 underwent purification on Sephadex and semi-prep HPLC with a MeOH and DIW mixture (1:1, isocratic) to yield compound **14** (5.8 mg).

Fraction IX (7.30 g) was chromatographed on silica gel using a hexane:EtOAc gradient (80:20 to 0:100), and sub-fraction 6 underwent purification on Sephadex and semi-prep HPLC with a MeOH and DIW mixture (1:1, isocratic) to yield compound **19** (300.8 mg).

Fraction VIII (5.5 g) underwent chromatography on silica gel. Subfractions 4, 5, 8, and 11 were subsequently chromatographed separately on Sephadex and semi-prep HPLC, following the previously mentioned protocol. This led to the isolation of compound **13** (8.4 mg) from sub-fraction 4, the compounds **6** (5.4 mg), **11** (10.8 mg), **12** (5.3 mg), and **18** (9.8 mg) from sub-fraction 5; compounds **5** (10.2 mg) and **15** (5.8 mg) from sub-fraction 8; and compounds **21** (4.4 mg) and **22** (25.2 mg) from sub-fraction 11. 

### 4.3. Compound ***11***

Whitish yellow powder ${\left[\alpha \right]}_{25}^{D}$–18.5 (c 0.01, MeOH); UV (MeOH) λ_max_ 315, 277; FTIR (film) 3378, 1677, 1605, 1500, and 1463 cm^−1^; ^1^H NMR (DMSO-*d_6_*, 400 MHz), and ^13^C NMR (DMSO-*d*_6_, 100 MHz), see Table 1; C_20_H_18_O_5_ by its HRFABMS (positive mode). HRFABMS *m*/*z* 339.1232 [M+H]+ (calcd for C_20_H_19_O_5_, 339.1227).

### 4.4. Cell Culture and Treatments

The human neuroblastoma SH-SY5Y cells were generously donated by the Blackburn Laboratory, University of Cape Town. Cells were grown in Dulbecco’s Modified Eagle’s medium (DMEM, Gibco, Life Technologies Corporation, Paisley, UK), supplemented with 10% fetal bovine serum (FBS, Gibco, Life Technologies Corporation, Paisley, UK), 100 U/mL penicillin, and 100 µg/mL streptomycin (Lonza Group Ltd., Verviers, Belgium). Cultures were incubated at 37 °C in humidified air with 5% CO_2,_ and cell growth medium was routinely changed every three days. Cells were sub-cultured when they attained 70 to 80 percent confluency, using a solution of 0.25% trypsin EDTA (Lonza Group Ltd., Verviers, Belgium).

### 4.5. Treatments

Stock solutions of 40 mg/mL and 10 mg/mL for total extract and compounds, respectively, were prepared in dimethyl sulfoxide (DMSO) (Sigma-Aldrich, St. Louis, MO, USA), from which final concentrations were made in cell growth media. To determine the optimum concentrations of licorice TE and compounds to be used for neuroprotection studies, SH-SY5Y cells were plated at a density of 10,000 cells/well and treated with concentrations of 12.5, 25, and 50 µg/mL of the TE, and 2.5, 5, and 10 µg/mL of the compounds. The vehicle-treated cells (cells treated with the same concentration of DMSO, similar to that of the highest concentration of extract) were used as control, and the treatments lasted 24 h. For MPP^+^, 2000 mM was chosen as the concentration used to establish neurotoxicity, which is in accordance with our published works [27,53]. For neuroprotection experiments, cells were plated as above and pre-treated with optimized concentrations of licorice TE and compounds for 2 h prior to the addition of 2000 µM MPP^+^. Treatments were incubated for 24 h, and the vehicle-treated cells served as control. 

### 4.6. Cell Viability Assays 

The MTT (Sigma-Aldrich, St. Louis, MO, USA) cell viability assay was used to determine the viability of cells following treatment with licorice TE- and compounds-only and the pre-treatment of cells with licorice TE and/or compounds and MPP^+^. Cells were seeded in 96-well plates and treated as stated above, after which the MTT assay was performed. After treatment, 10 or 20 µL (depending on well volume) of the 5 mg/mL MTT solution in phosphate-buffered saline (PBS) (Lonza Group Ltd., Verviers, Belgium) was added to each well and left to incubate in the dark at 37 °C for 4 h. After incubation, the medium containing the MTT dye was discarded, and the MTT formazan was solubilized with 100 μL of DMSO for an absorbance reading using a microplate reader (BMG Labtech Omega^®^ POLARStar, Offenburg, Germany) at a wavelength of 570 nm. Cell viability was calculated and expressed as a percentage of control. 

### 4.7. Adenosine Triphosphate Assay

The Mitochondrial ToxGlo ATP assay kit (Promega, Madison, WI, USA) was used to investigate cell ATP levels. Briefly, cells were plated at a density of 10,000 cells per well in a white 96-well plate, and after attachment, cells were treated as per the neuroprotection assay above. After treatment, cells were processed according to the manufacturer’s protocol by adding a volume of ATP detection reagent appropriate to the contents of each well and leaving the resulting mixture to incubate at 37 °C for 30 min. After incubation, luminescence intensity was read using the Promega GloMax^®^ explorer multimode microplate reader, and luminescence values were expressed as percentages of control.

### 4.8. Caspase 3/7 Apoptosis Assay

To investigate apoptosis in the cells, the Caspase 3/7 assay kit (Promega, Madison, WI, USA) was used to estimate levels of caspase 3/7 activity in the cells, in a manner according with the manufacturer’s instructions. Briefly, cells were plated in a white 96-well plate at a density of 10,000 cells per well and allowed to attach overnight, after which cells were pre-treated with licorice TE and compounds before the addition of 2000 µM MPP^+^. Treatments lasted for 24 h, and at the end of the experiments, equal volumes of Caspase 3/7 assay mix were added to each well. The luminescence intensity was read with the Promega GloMax → explorer multimode microplate reader. Luminescence values of the treated cells were expressed as a multiple of the control.

### 4.9. Statistical Analysis

Data generated from this study were analyzed using GraphPad Prism 6 and expressed as means and standard error values of means of three independent experiments performed in quadruplicate wells. One-way analysis of variance (ANOVA) was used to compare treated cells to either control or MPP+ alone. The significance of difference *p* < 0.05 was determined using Tukey’s multiple comparisons test and was indicated with * when comparing treatments to control or Φ when comparing licorice and TE and compounds to MPP^+^ treated cells.

## 5. Conclusions

This study investigated the neuroprotective potentials of *G. glabra* TE and isolated compounds in an in vitro model of PD. The results show that some of the compounds were cytotoxic (including glabrol (**6**), abyssinone (**5**), liquiritin apioside (**4**), glabrocoumarin (**12**), isomedicarpin (**14**), and 3,11-dioxooleana-1,12-die-29-oic acid (**21**)), which might have implications for the treatment of cancer. However, some of the compounds (naringenin 4′-*O*-glucoside (**1**), isoliquiritin (**7**), 3′[*O*],4′-(2,2-dimethylpyrano)-3,7-dihydroxyflavanone (**11**), ononin (**16**), and neoliquiritin (**20**)) were further studied for their neuroprotective potentials. The protective effects were related not only to the inhibition of ATP degeneration, but also to the attenuation of MPP+-induced elevated caspase 3/7 activities. These results indicate that the compounds isolated from *G. glabra* may provide novel therapeutic strategies for the treatment of PD. One limitation of this study was that the biological characterization was only performed on cell lines; therefore, in the future, it would be interesting to see if these compounds would have similar effects on in vivo models of PD. In addition, we recommend that future studies further elucidate the mechanisms of action, with emphasis on the molecular and genetic events underpinning the actions of these compounds. 

## Figures and Tables

**Table 1:** NMR data of compound **11** (DMSO-*d_6_*) and 3,4′,7-trihydroxy-3′-prenylflavanone (acetone-*d_6_*).

Position	Compound 11		3,4′,7-Trihydroxy-3′-prenylflavanone [51]	
	δ_C_	δ_H_, *multi, J* (Hz)	δ_C_	δ_H_, *multi, J* (Hz)
2	83.7	4.92 (*d*, 11.8)	84.6	5.03 (*d*, 11.9)
3	72.9	4.50 (*d*, 11.8)	73.4	4.59 (*d*, 11.9)
4	192.7	-	192.7	-
5	128.6	7.72 (*d*, 8.6)	129.3	7.74 (*d*, 8.6)
6	111.5	6.51 (*dd*, 8.6, 2.0)	111.2	6.64 (*dd*, 8.6, 2.2)
7	162.7	-	165.4	-
8	102.5	6.30 (*d*, 2.0)	103.1	6.41 (*d*, 2.2)
9	163.2	-	164.1	-
10	111.9	-	112.5	-
1′	129.6	-	128.9	-
2′	126.2	7.23 *(br s*)	130.0	7.35 (*d*, 2.0)
3′	120.7 s	-	128.1	-
4′	152.6	-	155.8	-
5′	115.5	6.76 (*d*, 7.9)	115.0	6.90 (d, 8.2)
6′	129.1 d	7.25 (*br d*, 7.9)	127.1	7.27 (*dd*, 8.2, 2.2)
1″	121.6	6.43 (*d,* 9.7)	28.7	3.38 (2H, *d*, 7.3)
2″	131.3	5.58 (*d*, 9.7)	123.1	5.39 (*m*)
3″	76.3	-	132.0	-
4″	27.7	1.37 (*s*)	25.4	1.72 (*br s*)
5″	27.7	1.37 (*s*)	17.4	1.74 (*br s*)

multi: multiplicity, *J* values in Hz, *s*: singlet, *br s*: broad singlet, *br d*: broad doublet, *d*: doublet, *dd*: doublet of doublet, *m*: multiple.

## Data Availability

The data associated with the isolated compounds, ^1^H NMR, ^13^C NMR spectra, and the biological data used to support the findings of this study are available from the corresponding author upon request.

## Supplementary Materials

The following supporting information can be downloaded at: https://www.mdpi.com/article/10.3390/ph17070852/s1, Table S1. ^1^H and ^13^C spectral data of compounds **1**-**22**, Figure S1. ^1^H and ^13^C spectra of compounds **1**-**22** and Scheme S1. The details of the isolation processes for compounds **1**−**22** from licorice.
