# Supplementary material for: Neuroprotective Effects of Glycyrrhiza glabra Total Extract and Isolated Compounds
Source: Pharmaceuticals (Basel). 2024 Jun 28;17(7):852. doi: 10.3390/ph17070852 (PMC11279424; doi:10.3390/ph17070852)

Neuroprotective effects of *Glycyrrhiza glabra* total extract and isolated compounds

Ali O.E. Eltahir[1a], Sylvester I. Omoruyi[2], Tanya N. Augustine[2], Robert C. Luckay[3], and Ahmed A. Hussein[1*]

Supplementary information

Table S1. ^{1}H and ^{13}C spectral data of compounds **1-22**

Figure S1. ^{1}H and ^{13}C spectra of compounds **1-22**

Scheme S1. The details of the isolation processes for compounds **1−6** from licorice

Table S1; ^{1}H- and ^{13}C-NMR data (400 MHz, DMSO-d_6) for compounds **1−22** isolated from *G. glabra*

**Name**	Naringenin 4′-*O*-glucoside **(1)**		3′, 4′ ,7-trihydroxyflavanone (butin) **(2)**		Liquiritin **(3)**	
Position	**δ_C**	**δ_H *(multi, J)***	**δ_C**	**δ_H *(multi, J)***	**δ_C**	**δ_H *(multi, J)***
2	78.45	5.50 (*dd*, 12.7, 2.2)	79.39	5.35 (*br d*, 12.2)	78.6	5.52 (*br d,*13.0)
3	42.45	3.24*<br>2.69 (*dd,* 17.1, 2.2)	43.75	3.03 (*dd*, 16.4, 12.2)<br>2.59 (*br d*, 16.4)	43.2	3.12 (*dd,* 16.6, 13.0)<br>2.66 (*br d*, 16.6)
4	196.01	-	190.39	-	189.9	-
5	163.16	-	128.72	7.60 (d, 7.2)	128.4	7.63 (*d*, 8.6)
6	95.87	5.84 (*br s*)	111.35	6.48 (*br s*)	110.8	6.36 (*dd,* 8.6, 2.0)
7	163.98	-	163.66	-	165.2	-
8	96.69	5.84 (*br s*)	103.06	6.30 (*br s*)	102.6	6.51 (*d,* 2.0)
9	163.16	-	163.66	-	163.1	-
10	101.71	-	113.46	-	113.3	-
1′	132.51	-	130.38	-	132.8	-
2′	128.51	7.43 (*d*, 8.6)	114.81	6.89 (*br s*)	128.0	7,44 (*d*, 8,6)
3′	116.63	7.07 (*d*, 8.2)	145.73	-	116.2	7.06 (*d*, 8.5)
4′	157.96	-	146.16	-	157.4	-
5′	116.62	7.07 (*d*, 8.2)	115.86	6.74 (*d*, 7.2)	116.2	7.06 (*d*, 8.5)
6′	128.51	7.43 (*d*, 8.6)	118.24	6.74 (*br d*, 7.2)	128.0	7,44 (*d*, 8.6)
1″	100.72	4.89 (*d,* 7.2)	-	-	100.3	4.89 (*d*, 7.3)
2″	73.67	3.24*	-	-	73.2	3.26*
3″	77.07	3.26*	-	-	77.0	3.33*
4″	70.14	3.17 (*dd*, 6.5, 10.5)	-	-	69.7	3.17 (*dd*, 6.0, 10.0)
5″	77.51	3.30*	-	-	76.6	3.28*
6″	61.12	3.68 (*d*, 11.0)<br>3.48*	-	-	60.7	3.69 (*d*, 11.4)<br>3.47 (*dd,* 5.5, 11.4)

** Overlapped or partially overlapped signals*

Cont. Table S1

	liquiritin apioside (**4**)		Abyssinone (**5**)		Glabrol (**6**)	
Position	**δ$_C$**	**δ$_H$ *(multi, J)***	**δ$_C$**	**δ$_H$ *(multi, J)***	**δ$_C$**	**δ$_H$ *(multi, J)***
2	78.9	5.51 (*dd*, 13.0, 2.2)	79.5	5.39 (*dd*, 12.5, 2.6)	72.7	4.95 (*br t*, 7.0)
3	43.1	3.11 (*dd,* 16.2, 13.0)<br>2.26 (*br d*, 16.2)	43.6	3.11 (*dd*, 16.5, 12.6)<br>2.60 (*dd*, 16.5, 2.6)	40.5	2.88 (*dd*, 14.0, 5.7)<br>2.69 (*dd*, 14.0, 7.1)
4	189.9	-	190.5	-	204.6	-
5	128.1	7.64 (*d*, 8.2)	128.8	7.63 (*d*, 8.5)	129.9	7.71 (*d*, 8.7)
6	110.8	6.51 (*dd*, 8.2,2.1)	111.0	6.49 (*dd*, 8.8, 2.0)	107.5	6.44 (*d*, 8.7)
7	165.1	-	165.5	-	162.7	-
8	102.6	6.35 (*d*, 2.1)	103.0	6.31 (*d*, 2.0)	114.3	-
9	163.1	-	163.6	-	162.8	-
10	113.3	-	113.7	-	110.5	-
1′	132.4	-	129.7	-	127.7	-
2′	128.4	7.44 (*d*, 8.5)	128.6	7.16 (*br s*)	114.4	6.65 (*br s*)
3′	116.0	7.04 (*d*, 8.5)	127.8	-	126.8	-
4′	157.3	-	155.7	-	153.7	
5′	116.0	7.04 (*d*, 8.5)	115.1	6.80 (*d*, 8.5)	130.3	6.82 (*d*, 9.0)
6′	128.1	7.44 (*d*, 8.5)	125.9	7.14 (dd, 8.5, 1.93)	127.4	6.84 (*br d*, 9.0)
1″	98.6	4.96 (*d*, 7.2)	27.9	3.22 (*d*, 6.5, 2H)	21.2	3.11 (*d*, 6.8)
2″	75.7	3.49*	123.1	5.28 (*br d*, 6.5)	122.33	5.15 (*t*, 6.8)
3″	76.9	3.36*	131.7	-	130.44	-
4″	70.0	3.17 (*t*, 9.2)	26.0	1.68 (*s*)	25.5*	1.65* (*s*)
5″	76.1	3.75 (s)	18.1	1.67 (*s*)	25.4*	1.62* (*s*)
6″	60.6	3.45**<br>3.69 (*br d*, 11.6)	-	-	-	-
1‴	108.7	5.36 (*s*)	-	-	28.02	3.18 (*d*, 6.8)
2‴	77.0	3.48**	-	-	122.9	5.20 (*t*, 6.8)
3‴	79.3	-	-	-	126.8	-
4‴	74.0	3.65 (*d*, 9.3)<br>3.94 (*d*, 9.3)	-	-	17.6**	1.71** (*s*)
5‴	64.3	3.33* (2H)	-	-	17.5**	1.63** (*s*)

*,**Overlapped or partially overlapped signals

Cont. table S1

	Isoliquiritin (**7**)		Neoisoliquiritin (**8**)		Isoliquiritin apioside (**9**)		Licuraside (**10**)	
Position	**δ$_C$**	**δ$_H$ *(multi, J)***	**δ$_C$**	**δ$_H$ *(multi, J)***	**δ$_C$**	**δ$_H$ *(multi, J)***	**δ$_C$**	**δ$_H$ *(multi, J)***
C=O	191.7	-	192.5	-	191.1	-	192.07	-
ά	119.6	7.87 (*d,* 15.5)	117.7	7.82 (*d*, 16.6)	119.2	7.86 (*d*, 15.8)	117.30	7.83 (*d*, 16.0)
β	143.8	7.77 (*d*, 15.5)	145.5	7.79 (*d*, 16.6)	143.2	7.78 (*d*, 15.8)	145.05	7.80 (*d*, 16.0)
1	133.4	-	125.9	-	128.4	-	125.59	-
2	128.8	7.86 (*d,* 8.8)	132.9	7.79 (*d*, 8.4)	130.7	7.86 (*d*, 8.8)	131.43	7.78 (*d*, 8.8)
3	116.9	7.10 (*d*, 8.8)	116.4	6.85 (*d*, 8.4)	116.3	7.07 (*d*, 8.8)	115.89	6.85 (*d*, 8.8)
4	159.9	-	161.2	-	159.2	-	160.58	
5	116.9	7.10 (*d*, 8.8)	116.4	6.85 (*d*, 8.4)	116.3	7.07 (*d*, 8.8)	115.89	6.86 (*d*, 8.8)
6	128.8	7.86 (*d*, 8.8)	131.9	7.87 (*d*, 8.4)	130.7	7.86 (*d*, 8.8)	131.43	7.78 (*d*, 8.8)
1′	113.4	-	115.2	-	112.6	-	114.83	-
2′	166.3	-	163.9	-	165.6	-	163.29	-
3′	103.0	6.25 (*d*, 1.7)	103.9	6.58 (*d*, 1.8)	102.6	6.25 (*d*, 2.0)	103.36	6.56 (*d*, 2.0)
4′	166.3	-	165.6	-	165.6	-	165.6	-
5′	108.9	6.41 (*dd*, 1.7, 9.0)	108.6	6.62 (*dd*, 1.8, 9.0)	108.6	6.25 (*d*, 2.0)	108.72	6.59 (*dd*, 9.0, 2.0)
6′	131.2	8.18 (*d*, 9.0)	131.9	8.27 (*d*, 9.0)	132.9	8.17 (*d*, 9.0)	133.3	8.28 (*d*, 9.0)
1″	100.3	4.98 (*d,* 8.8)	100.0	5.04 (*d*, 9.0)	98.3	5.05 (*d*, 7.4)	97.86	5.12 (*d*, 7.0)
2″	73.6	3.29 (*d,* 8.6)	73.5	3.24 (*d, 7.4*)	79.3	3.74 (*br s*)	77.00	3.44*
3″	77.0	3.30 (*t,* 8.6)	76.9	3.31 (*t, 9.0*)	76.9	3.49*	76.04	3.50*
4″	70.1	3.17 (*t,* 8.8)	70.0	3.17 (*t, 8.4*)	69.9	3.19 (*t*, 7.5)	69.82	3.18*
5″	77.6	3.34 (*dd*, 9.4, 7.6)	77.6	3.34 (*dd, 9.0, 7.0*)	77.1	3.42*	76.77	3.74 (*s*)
6″	61.1	3.69 (*d*, 11.0)	61.0	3.69 (*dd*, 11.0)	60.5	3.45*	60.63	3.69*
		3.18 (*dd*, 8.0, 11.0)		3.34 (*dd*, 11.0, 7.0)		3.70 (*br d,* 10.9)		3.46*
1‴	-	-	-	-	108.6	5.36 (*br s*)	108.06	5.34 (*s*)
2‴	-	-	-	-	76.0	3.51 (*d*, 7.4)	75.68	3.50*
3‴	-	-	-	-	79.3	-	79.28	-
4‴	-	-	-	-	74.0	3.65 (*d*, 9.5)	73.97	3.90 (*d*, 9.5)
						3.94 (*d*, 9.5)		3.65 (*d*, 9.5)
5‴	-	-	-	-	64.3	3.31 (*s*, 2H)	64.26	3.30*

*Overlapped or partially overlapped signals.

Cont. table S1

	3'[*O*],4'-(2,2-dimethylpyrano)-3,7-dihydroxyflavanone **(11)**		Glabrocoumarin **(12)**		Glabrene **(13)**	
Position	**δ_C**	**δ_H *(multi, J)***	**δ_C**	**δ_H *(multi, J)***	**δ_C**	**δ_H *(multi, J)***
2	83.7	4.92 (*d*, 11.8)	161.2	-	67.7	4.89 (2H, *s*)
3	72.9	4.50 (*d*, 11.8)	121.3	-	127.5	-
4	192.7	-	142.5	7.82 (*s*)	119.9	6.50 (*br s*)
5	128.6	7.72 (*d*, 8.6)	129.6	7.52 (*d*, 9.0)	127.5	6.92 (*d,* 7.8)
6	111.5	6.51 (*dd*, 8.6, 2.0)	113.1	6.79 (*br d*, 9.0)	108.6	6.33 (*br d*, 7.8)
7	162.7	-	160.3	-	158.1	-
8	102.5	6.30 (*d*, 2.0)	101.7	6.74 (*br s*)	102.4	6.24 (*br s*)
9	163.2	-	155.3	-	153.9	-
10	111.9	-	111.8	-	115.3	-
1′	129.6	-	116.4	-	117.6	-
2′	126.2	7.23 (*br s*)	150.55	-	150.6	-
3′	120.7	-	107.7	-	108.9	-
4′	152.6	-	153.3	-	153.1	-
5′	115.5	6.76 (*d,* 7.9)	107.3	6.32 (*d*, 7.4)	108.1	6.41 (*d*, 8.4)
6′	129.1	7.25 (*br d*, 7.9)	130.6	6.94 (*d*, 7.4)	127.8	7.00 (*d*, 8.4)
1″	121.6	6.43 (*d*, 9.7)	116.05	6.68 (*d*, 9.4)	116.8	6.57 (*d*, 10.0)
2″	131.3	5.58 (*d*, 9.7)	128.8	5.68 (*d*, 9.4)	128.3	5.53 (*d*, 10.0)
3″	76.3	-	75.4	-	76.11	-
4″- CH_3	27.7	1.37 (*s*)	27.4	1.35 (*s*)	27.2	1.37 (*s*)
5″- CH_3	27.7	1.37 (*s*)	27.4	1.35 (*s*)	27.2	1.37 (*s*)

Cont. table S1

	Isomedicarpin **(14)**		7-hydroxy-4′-methoxyflavone (formononetin) **(15)**		Ononin **(16)**		Glycyroside **(17)**	
	**δ$_C$**	**δ$_H$ *(multi, J)***	**δ$_C$**	**δ$_H$ *(multi, J)***	**δ$_C$**	**δ$_H$ *(multi, J)***	**δ$_C$**	**δ$_H$ *(multi, J)***
2	65.8	3.50*	153.0	8.32 (*s*)	154.1	7.0 (*d*, 8.4)	153.6	8.45 (*s*)
3	39.30	3.52*	124.2	-	124.4	-	124.0	-
4	78.0	5.47 (*d*, 6.7)	174.6	-	175.1	-	174.6	-
5	132.0	7.19 (*d*, 8.3)	127.2	7.50 (*d*, 8.6)	127.4	8.06 (*d*, 8.8)	127.0	8.05 (*d,* 8.8)
6	109.6	6.41 (*dd*, 8.3, 2.3)	115.4	6.93 (*br d*, 8.6)	116.1	7.0 (*d*, 8.4)	115.5	7.13 (*dd*, 2.0, 8.8)
7	160.5	-	163.1	-	161.9	-	161.2	-
8	102.8	6.19 (*d*, 2.3)	102.1	6.86 (*br s*)	103.8	7.24 (*s*)	103.3	7.22 (*d*, 2.0)
9	156.3	-	157.5	-	157.5	-	157.0	-
10	111.2	-	116.3	-	118.9	-	118.4	-
1′	119.3	-	123.57	-	123.8	-	123.3	-
2′	160.3	-	130.0	7.50 (*d*, 8.6)	130.5	7.54 (*d*, 8.4)	130.0	7.53 (*d*, 8.6)
3′	96.3	6.35 (*br s*)	113.5	6.99 (*d*, 8.6)	116.4	7.15 (*d*, 8.8)	113.6	7.00 (*d*, 8.8)
4′	158.7		158.7	-	159.5	-	159.0	-
5′	105.9	6.38 (*dd*, 8.0, 2.3)	113.5	6.99 (*d*, 8.6)	114.1	7.15 (*d*, 8.8)	113.6	7.00 (d, 8.8)
6′	125.1	7.16 (*d*, 8.0)	130.0	7.50 (*d*, 8.6)	127.4	7.54 (*d*, 8.4)	130.0	7.53 (d, 8.6)
1″	-	-	-	-	100.4	5.11 (*d*, 6.7)	98.3	5.20 (*d*, 7.4)
2″	-	-	-	-	73.5	3.30*	76.0	3.74 (*br s*)
3″	-	-	-	-	76.9	3.31*	75.6	3.54 (*d*, 7.6)
4″	-	-	-	-	70.0	3.20*	69.8	3.20 (*d*, 8.8)
5″	-	-	-	-	77.6	3.47 (*d*, 8.6)	77.0	3.19 (*d*, 8.2)
6″	-	-	-	-	61.0	3.47 (*d*, 8.6)<br>2.33 (*br s*)	60.5	3.49 (*d*, 8.0)<br>3.71 (*br s*)
1‴	-	-	-	-	-	-	108.7	5.35 (*br s*)
2‴	-	-	-	-	-	-	76.8	3.49 (*d*, 8.0)
3‴	-	-	-	-	-	-	79.3	-
4‴	-	-	-	-	-	-	73.9	3.93 (*d*, 9.2)<br>3.66 (*d*, 9.2)
5‴	-	-	-	-	-	-	64.1	3.26 (*br s*)<br>3.20 (*d*, 8.8)
OCH_3	55.2	3.65 (*s*)	55.1	3.79 (*s*)	55.60	3.78 (*s*)	55.16	3.74 (*br s*)

**Overlapped or partially overlapped signals*

Cont. table S1

	(3*S*)-7,4′-dihydroxy-2′-methoxyisoflavan **(18)**		glabridin **(19)** Acetone-*d6*		neoliquiritin **(20)**	
-	**δC**	**δH *(multi, J)***	**δC**	**δH *(multi, J)***	**δC**	**δH *(multi, J)***
1	-	-	-	-	-	-
2	69.2	3.61 (*t*, 9.9)<br>4.14 (*br d*, 9.9)	70.0	4.20 (*d*, 10.0)<br>3.85 (*d*, 10.0)	79.2	5.50 (*dt*, 13.5)
3	31.0	3.32 (*m*)	31.6	3.33 (*m*)	43.1	3.20*<br>2.67 (*d*, 16.5)
4	29.7	2.71 (*dd*, 15.7, 4.3)<br>2.87 (*dd*, 15.7, 11.3)	30.3	2.64 (*d*, 15.3)<br>2.84 (*dd*, 3.1, 15.3)	190.8	-
5	130.0	6.85 (*d*, 8.4)	127.8	6.15 (*d*, 8.2)	128.0	7.72 (*d*, 8.7)
6	107.9	6.29 (*br d*, 8.4)	109.5	6.69 (*d*, 8.2)	110.9	6.72 (*br d*, 8.7)
7	156.4	-	151.8	-	163.5	-
8	102.5	6.19 (*br s*)	106.8	-	103.4	6.66 (*br s*)
9	154.5	-	149.7	-	162.9	-
10	112.7	-	114.7	-	115.3	-
1′	119.7	-	118.3	-	129.03	-
2′	158.8	-	155.9	-	128.4	7.34 (*d*, 8.4)
3′	101.3	6.43 (*br s*)	102.6	6.33 (*d*, 2.3)	115.1	6.80 (*d*, 8.2)
4′	155.9	-	157.1	-	157.0	-
5′	104.3	6.34 (*br d*, 8.6)	108.3	6.14 (*dd,* 2.3, 8.4)	115.1	6.80 (*d*, 8.2)
6′	127.6	6.97 (*d*, 8.6)	128.7	6.81 (*d*, 8.4)	128.4	7.34 (*d*, 8.4)
1″	-	-	116.8	6.48 (*d*, 9.8)	99.7	4.99 (*t*, 7.5)
2″	-	-	129.2	5.47 (*d*, 9.8)	73.0	3.24*
3″	-	-	75.1	-	76.3	3.26*
4″	-	-	-	-	69.4	3.16*
5″	-	-	-	-	77.0	3.87*
6″	-	-	-	-	60.5	3.45*, 3.66*
OCH_3	54.8	3.66 (s)	26.6	1.23 (*s*)	-	-
OCH_3	-	-	26.6	1.23 (*s*)	-	-

** Overlapped or partially overlapped signals.*

Cont. S1

Position	AL-47-2; (21) 3,11-dioxooleana-1,12-dien-29-oic acid (400 MHZ, $CDCl_3$)		AL-47-3; (22) 3-oxo-glycyrrhetinic acid (400 MHZ, CDCl3)	
	**δ_C**	**δ_H *(multi, J)***	**δ_C**	**δ_H *(multi, J)***
1	161.6	7.72 (*d*, 10.4)	39.7	2.99(*ddd*, 14.4, 6.8, 4.8), 1.43*
2	124.5	5.83 (*d*, 10.4)	34.2	2.66 (*ddd*, 15.8, 11.0, 7.0), 2.38(*ddd*, 15.8, 6.5, 4.0)
3	204.6	-	217.5	-
4	44.7	-	47.8	-
5	52.7	1.54*	55.4	1.31*
6	18.1	1.61* (CH_2)	18.7	1.58* (CH_2)
7	30.8	1.99*, 1.37*	32.1	1.71*, 1.49*
8	45.5	-	45.2	-
9	55.5	2.66 (*s*)	61.0	2.46 (*s*)
10	38.7	-	36.7	-
11	199.0	-	199.7	-
12	128.1	5.78 (*s*)	128.4	5.77 (*s*)
13	170.6	-	169.8	-
14	43.4	-	43.3	-
15	26.5	1.21*, 1.85 (*ddd*, 17.0, 13.4, 4.0)	26.5	2.06[a] (*td*, 13.2, 4.5); 1.23[b]*
16	26.2	1.03*, 2.36*	26.5	1.89[a] (*td*, 13.2, 3.7); 1.06[b]*
17	31.8	-	31.8	-
18	48.3	2.22 (*dd*, 14.3, 3.3)	48.2	2.23 (*dd*, 13.6, 2.9)
19	40.8	1.63*, 1.93*	40.9	1.96 (*br d,* 13.6), 1.65 (*t*, 13.6)
20	43.7	-	43.2	-
21	32.0	1.47*, 1.70*	30.9	2.03 (*br d*, 10.0), 1.39*
22	37.6	1.42* (CH_2)	37.7	1.44* (CH_2)
23	27.5	1.14 (s, CH_3)	26.3	1.13 (s, CH_3)
24	21.5	1.09 (s, CH_3)	21.4	1.09 (s, CH_3)
25	20.0	1.39 (s, CH_3)	15.6	1.29 (s, CH_3)
26	18.9	1.16 (s, CH_3)	18.5	1.19 (s, CH_3)
27	23.4	1.37 (s, CH_3)	23.3	1.40 (s, CH_3)
28	28.5	0.85 (s, CH_3)	28.5	0.87 (s, CH_3)
29	28.3	1.21 (s, CH_3)	28.4	1.25 (s, CH_3)
30	180.9	-	181.1	-

[a,b] exchangeable protons. All these assignments were in agreement with COSY, HSQC and HMBC spectra. * Overlapped or partially overlapped signals; δ values were measured from the HSQC spectra.

7.5.1. Figure S1. ^{1}H and ^{13}C-NMR spectrum of compounds 1 -22 isolated from *G. glabra*

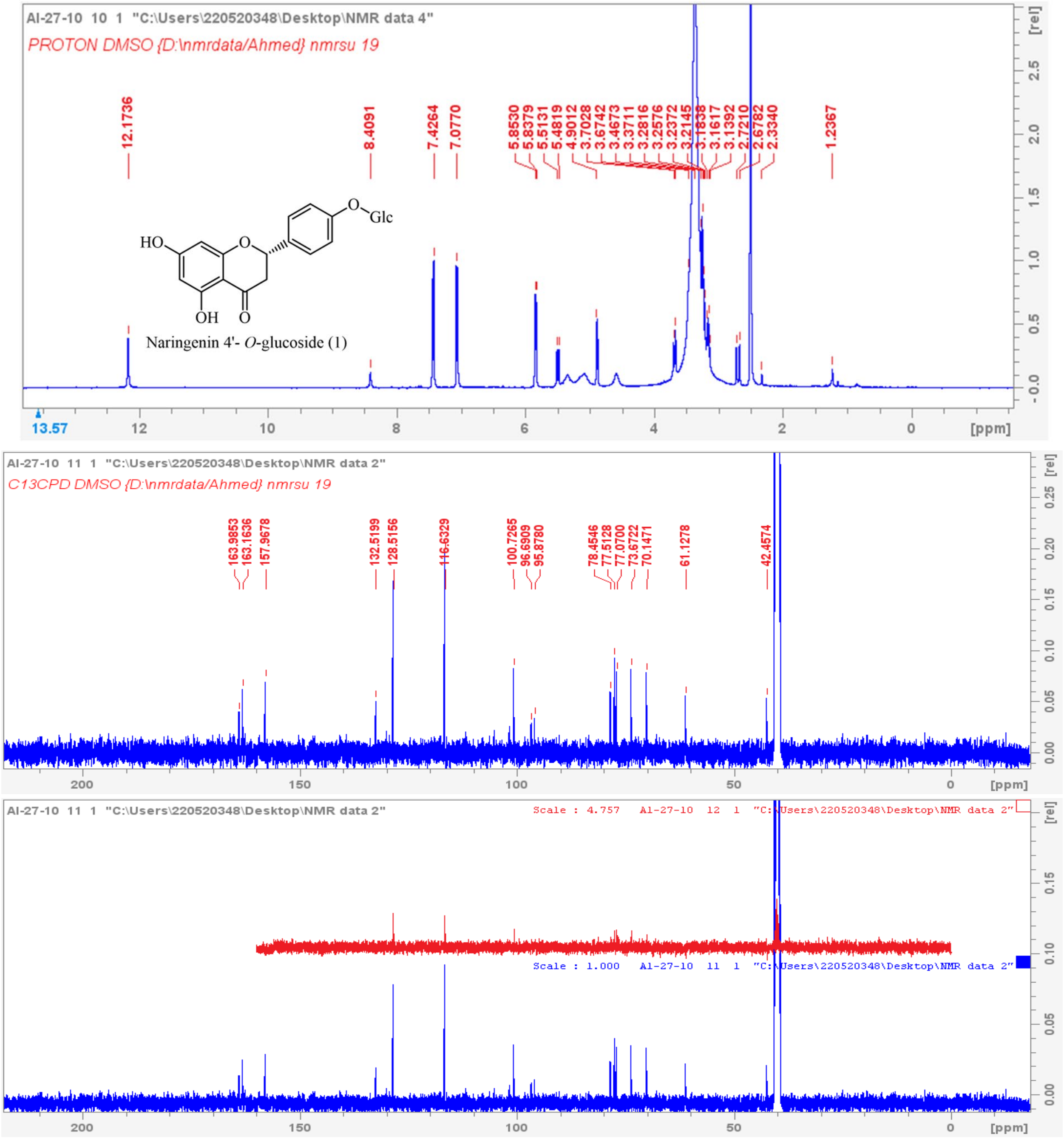

Figure S1. 1; ^{1}H and ^{13}C-NMR (400 MH_Z, DMSO-d_6) spectrum of compound **1**; (AL-27-10)

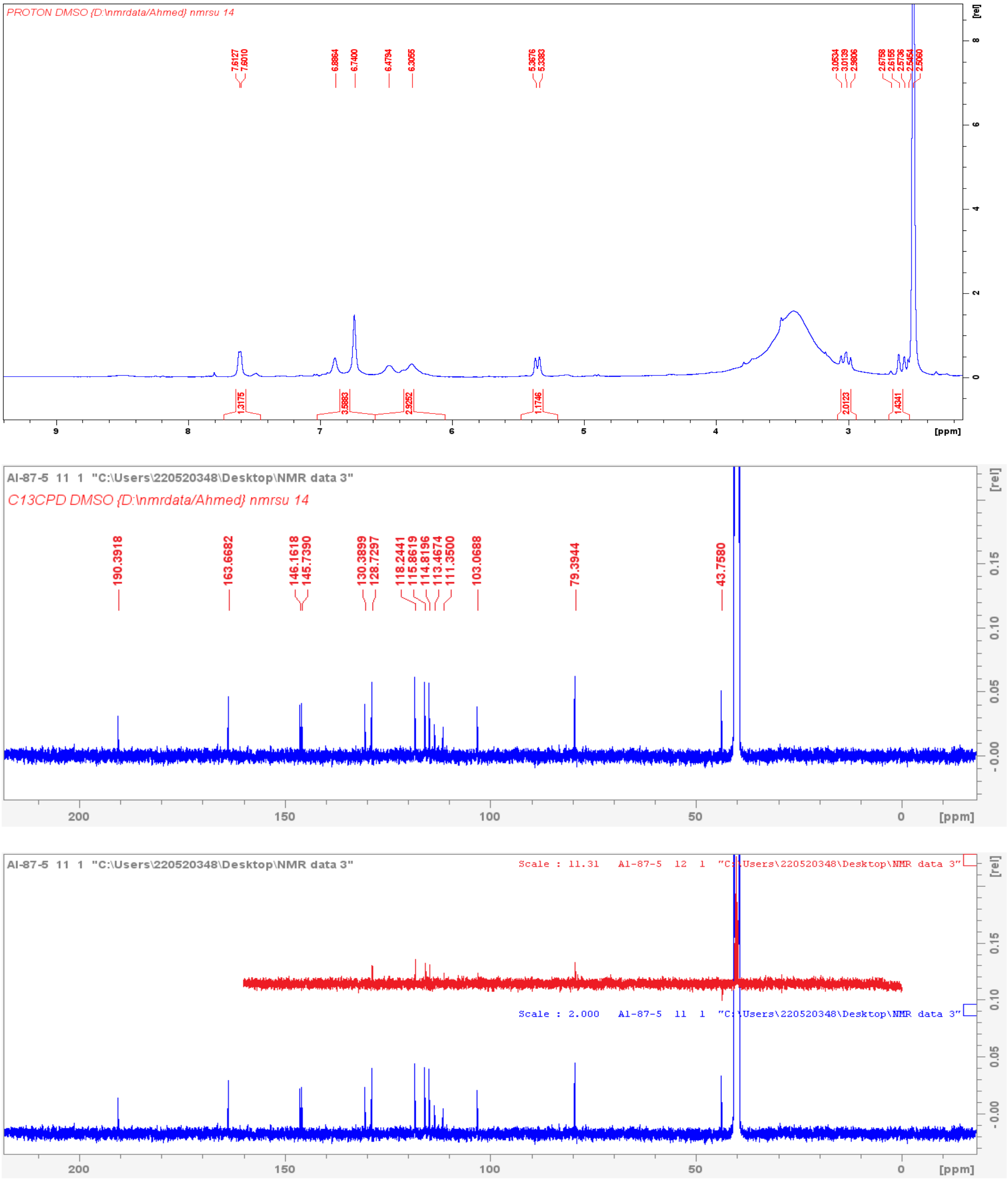

Figure S. 1. 2; ^{1}H and ^{13}C-NMR (400 MHz, DMSO-d_6) spectrum of compound **2**; (AL-87-5)

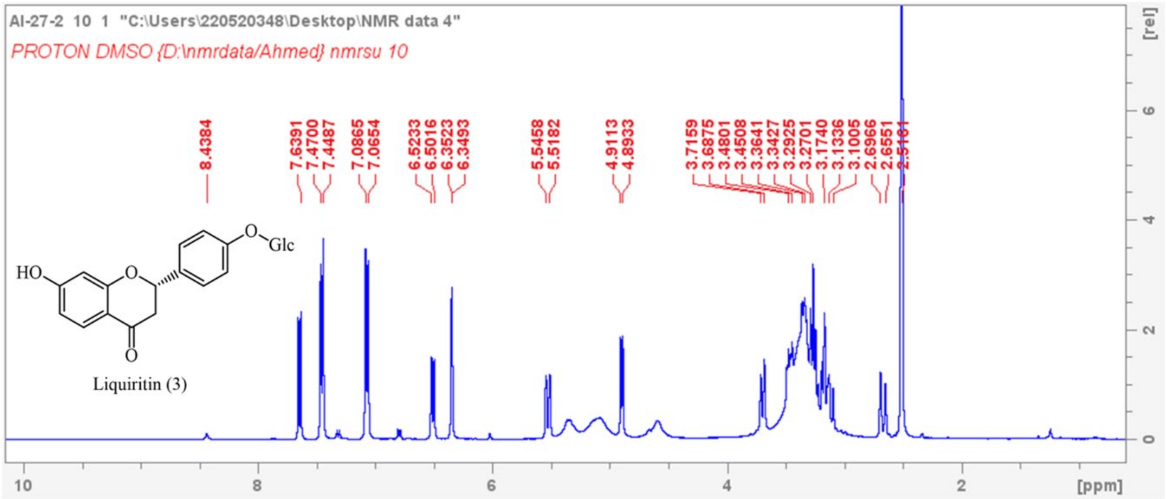

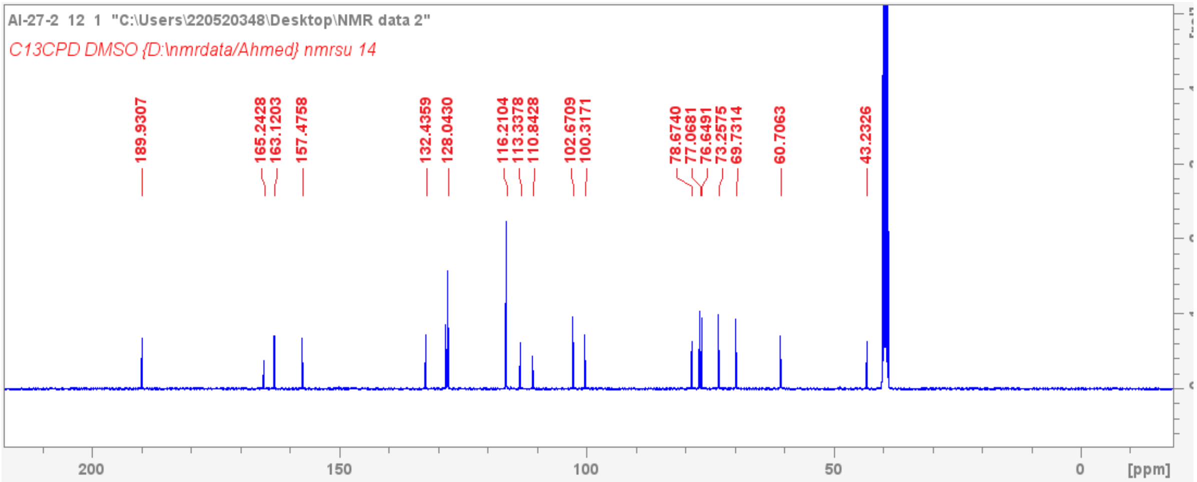

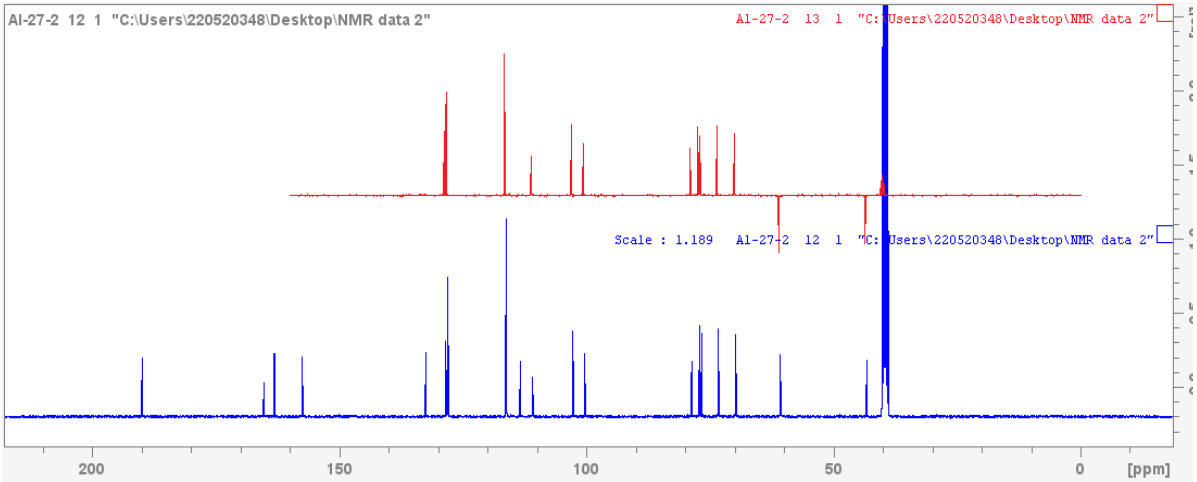

Figure S1. 3; ^{1}H and ^{13}C-NMR (400 MHz, DMSO-d_6) spectrum of compound **3**; (AL-27-2)

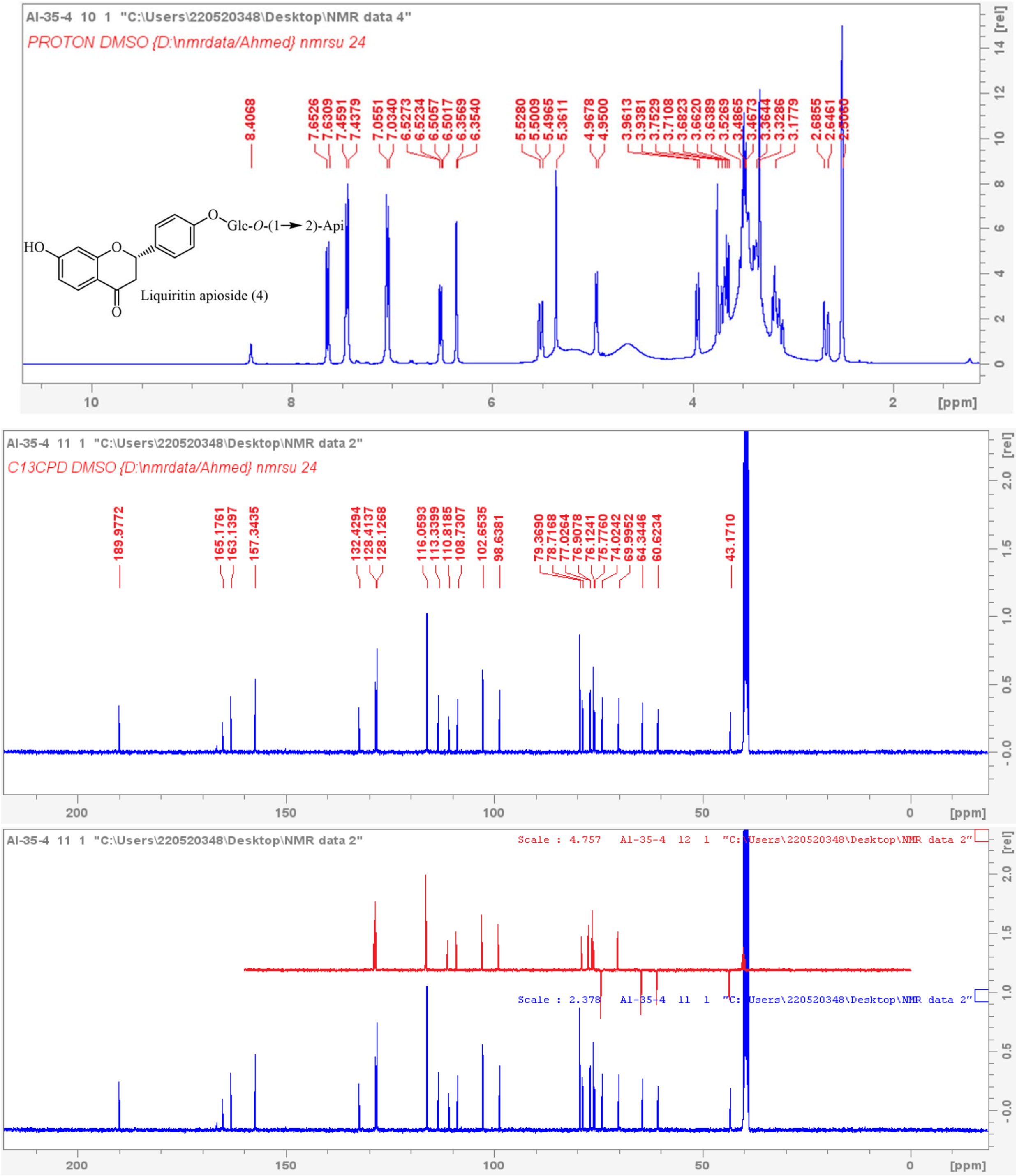

Figure S1. 4; ^{1}H and ^{13}C-NMR (400 MHz, DMSO-d_6) spectrum of compound **4**; (AL-35-4)

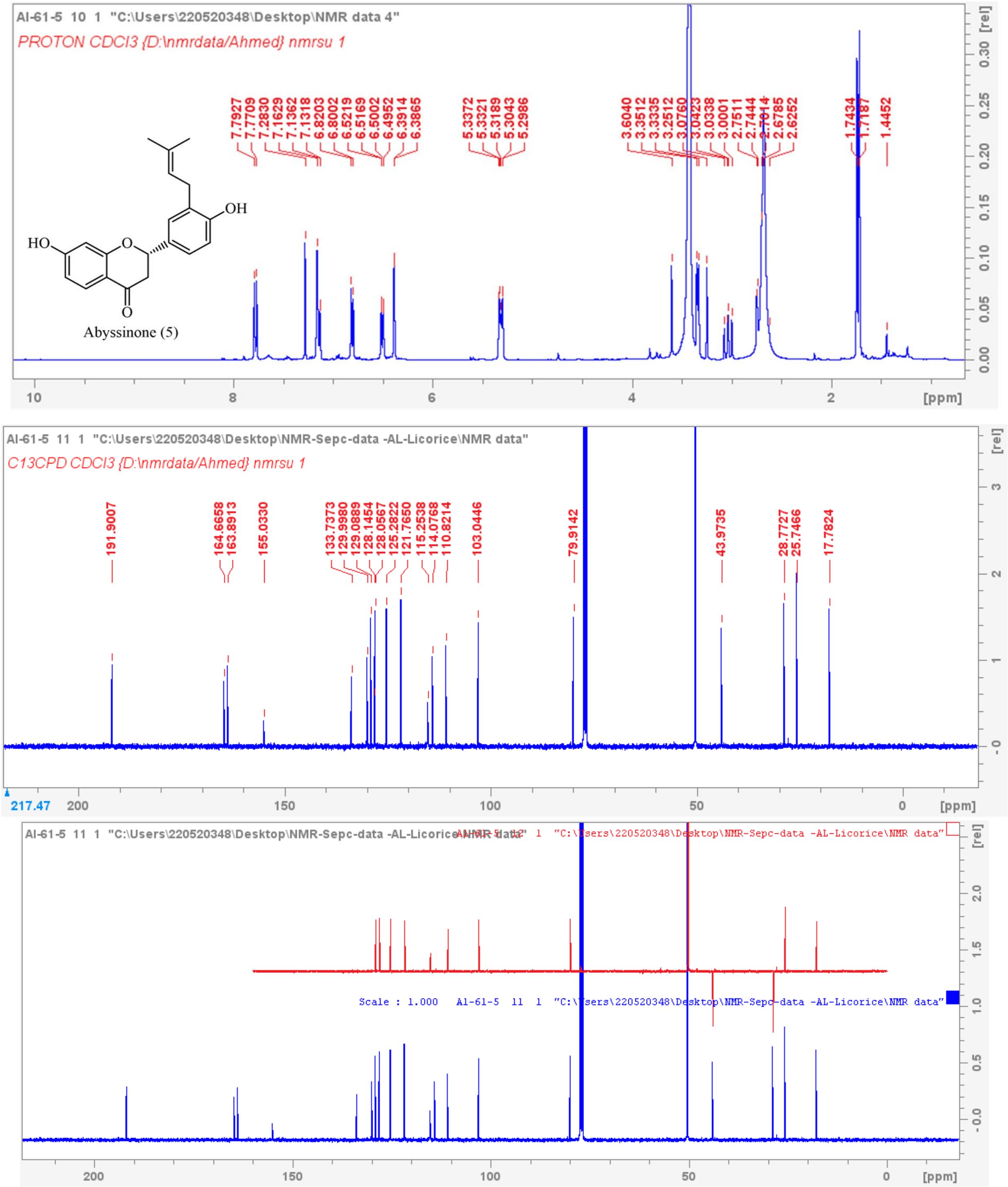

Figure S1. 5; ^{1}H and ^{13}C-NMR (400 MH_Z, CDCl3-d_6) spectrum of compound **5**; (AL-61-5)

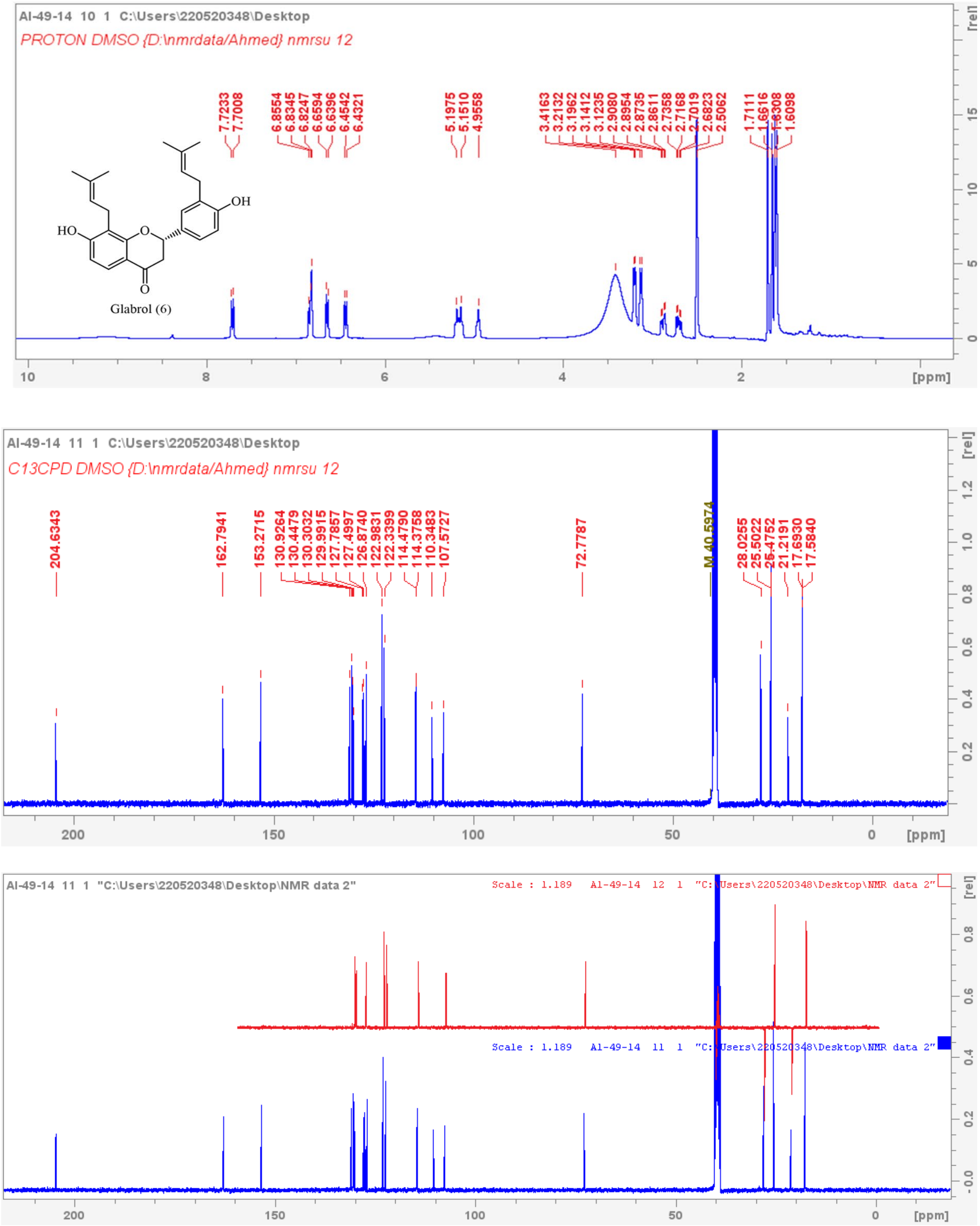

Figure S1. 6; ^{1}H and ^{13}C-NMR (400 MH$_Z$, DMSO-d_6) spectrum of compound **6**; (AL-49-14)

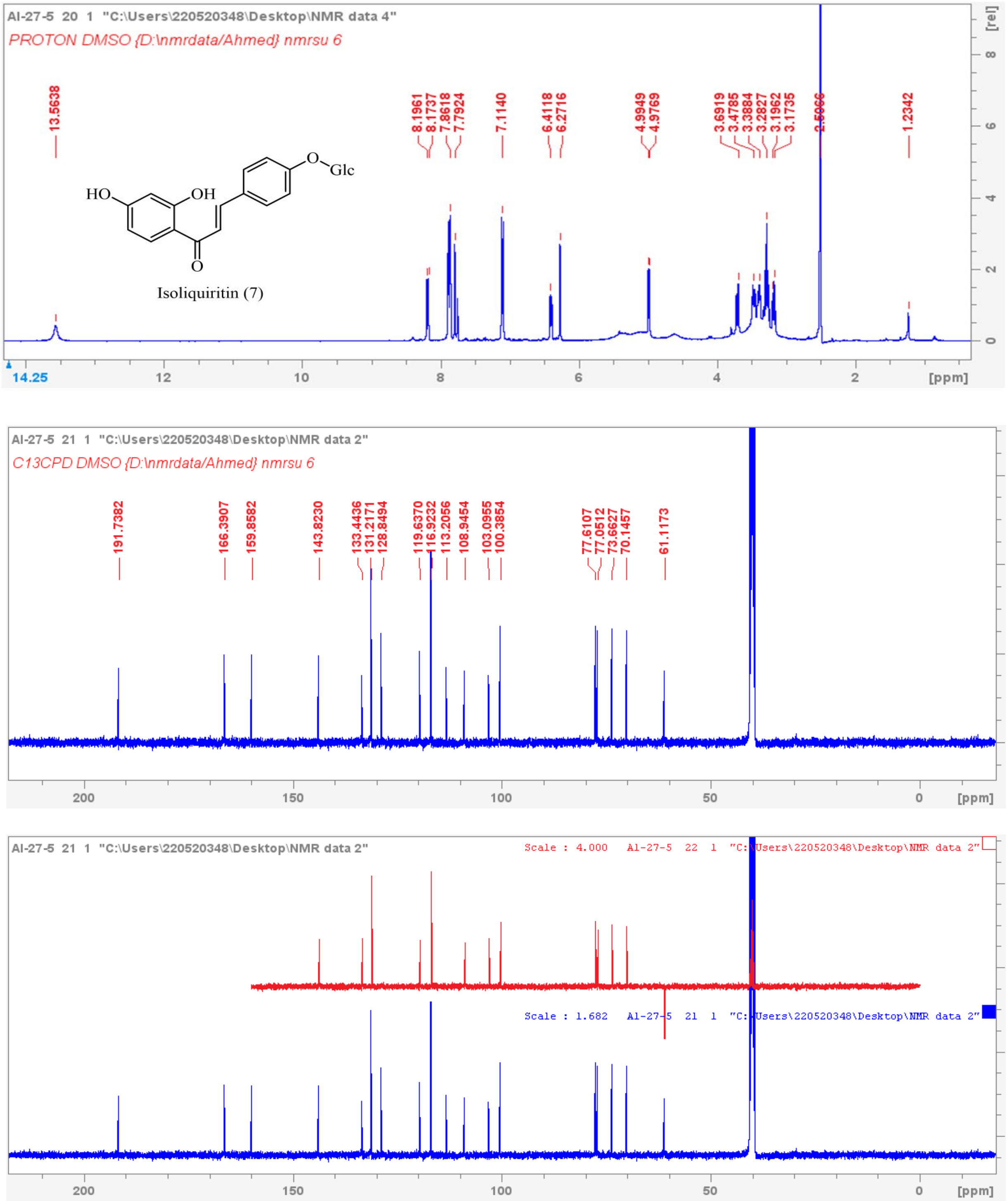

Figure S1. 7; ^{1}H and ^{13}C-NMR (400 MH_Z, DMSO-d_6) spectrum of compound **7**; (AL-27-5)

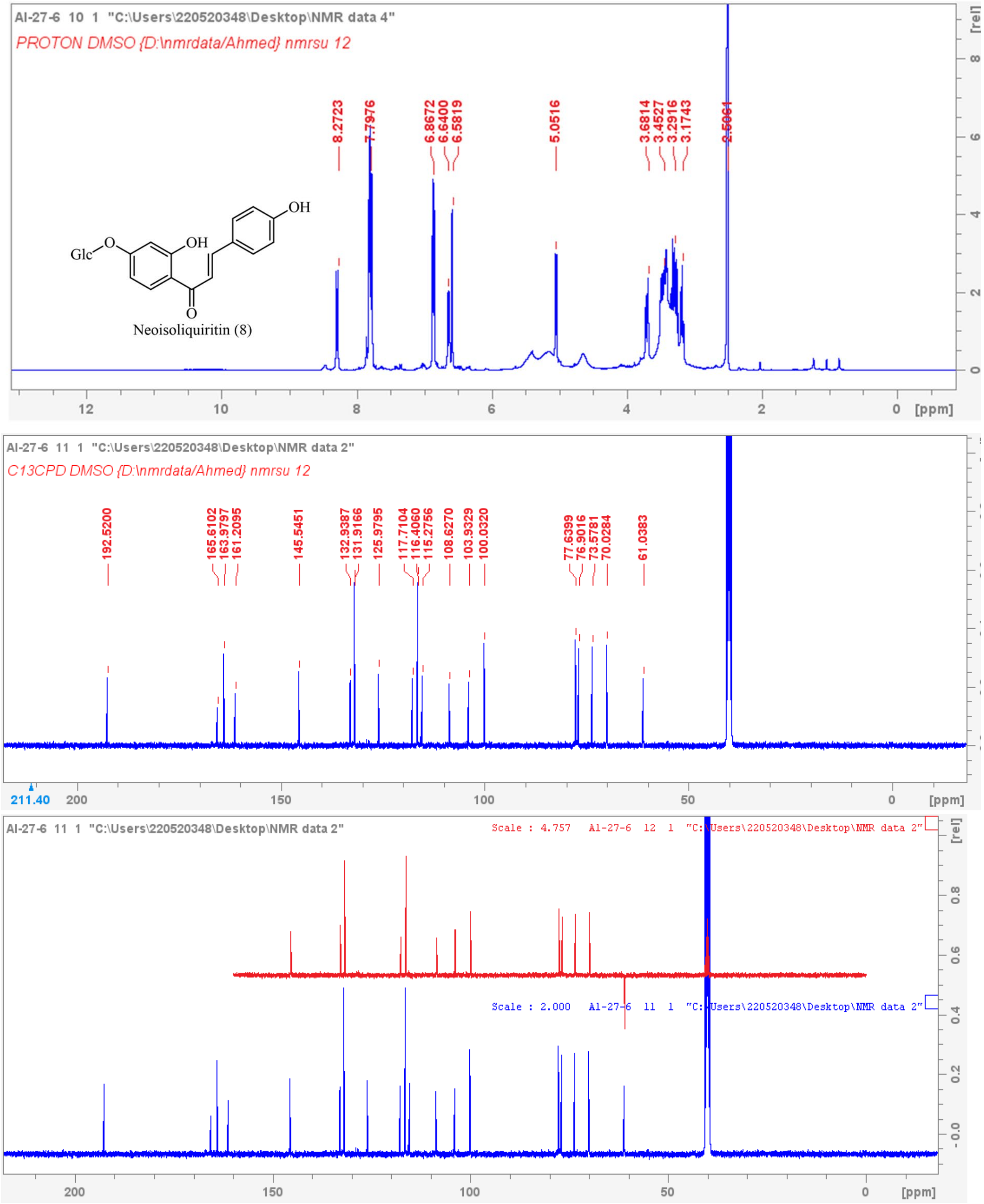

Figure S1. 8; ^{1}H and ^{13}C-NMR (400 MH$_{Z}$, DMSO-d_6) spectrum of compound **8**; (AL-27-6)

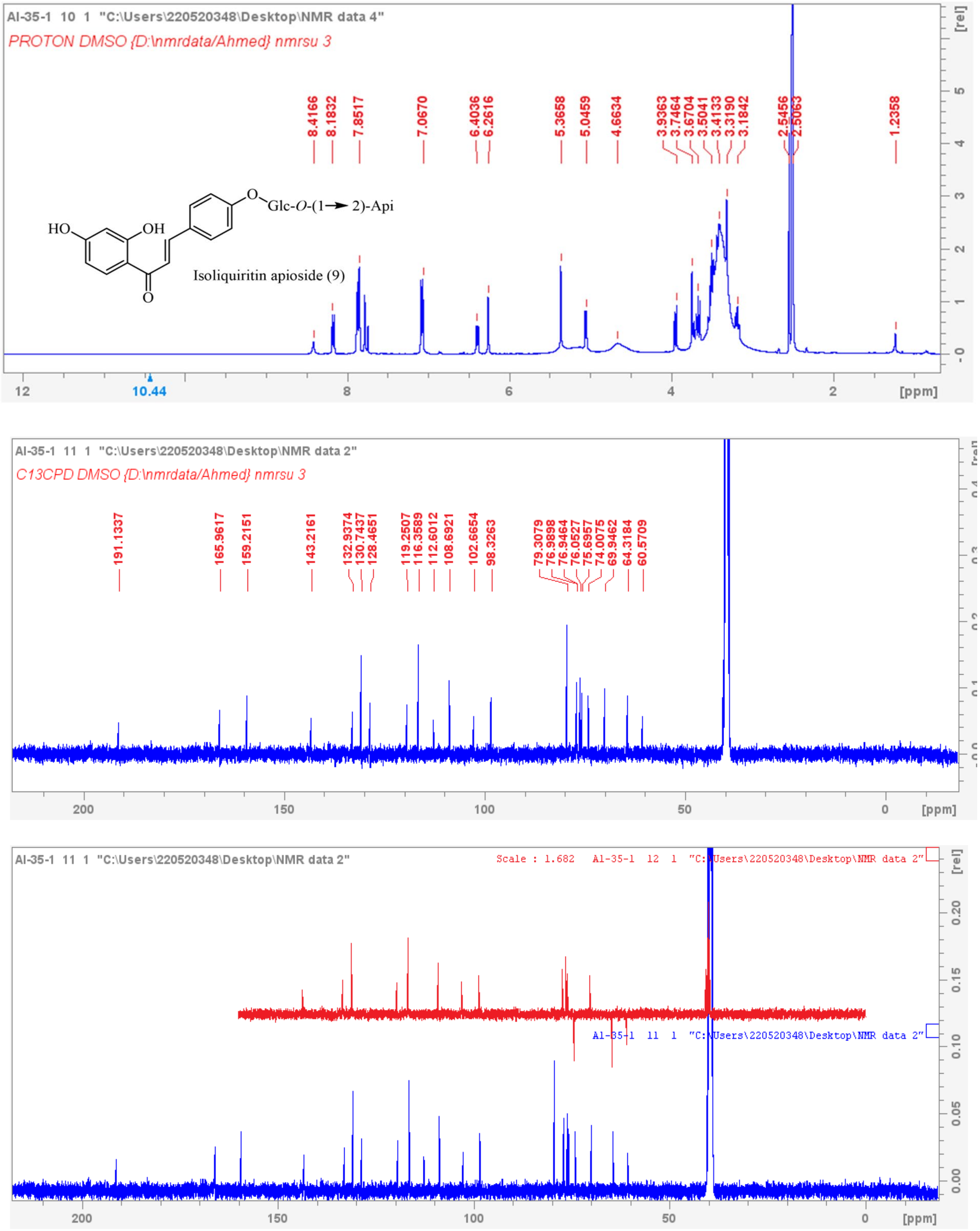

Figure S1. 9; ^{1}H and ^{13}C-NMR (400 MH_Z, DMSO-d_6) spectrum of compound **9**; (AL-35-1)

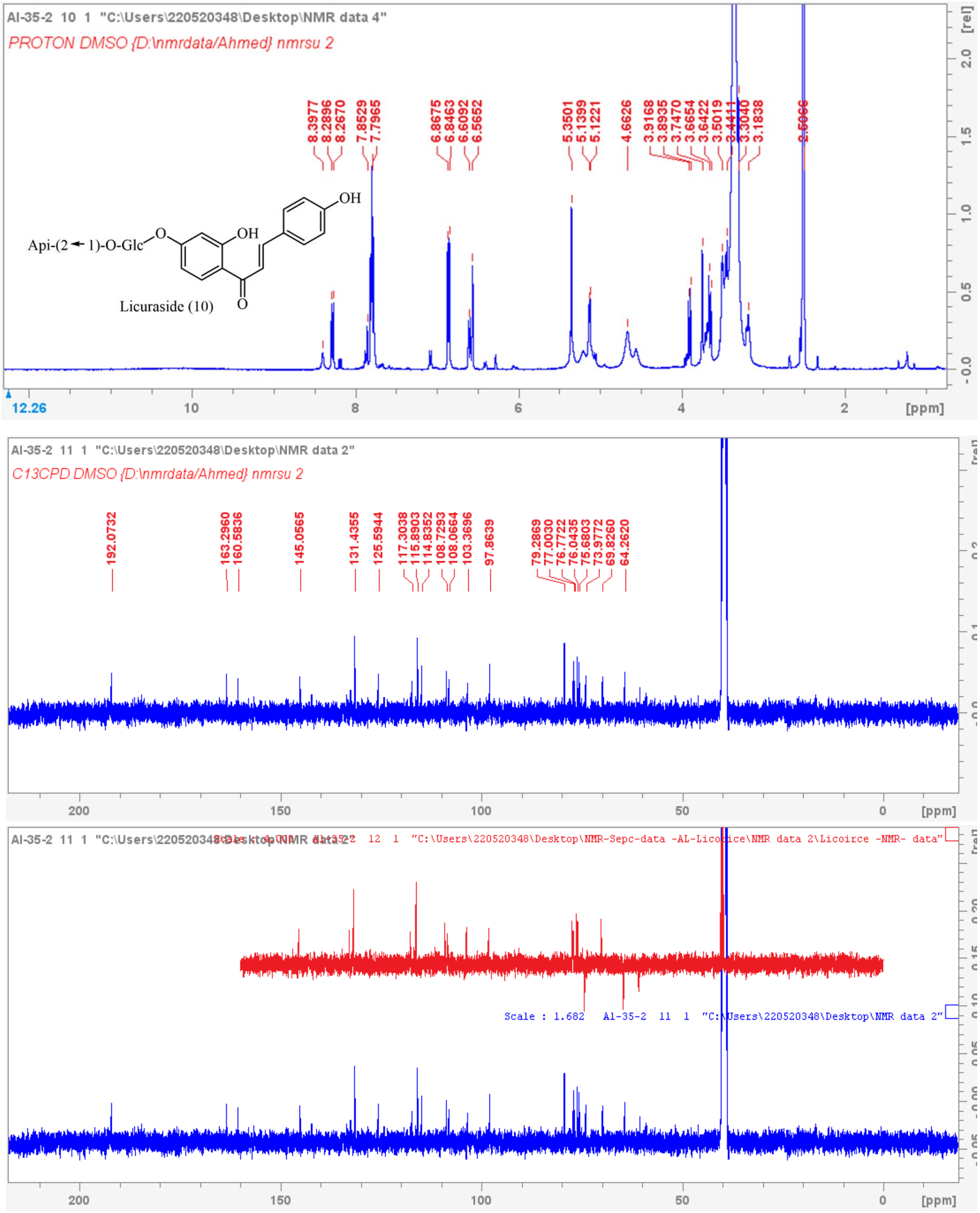

Figure S1. 10; ^{1}H and ^{13}C-NMR (400 MHz, DMSO-d_6) spectrum of compound **10**; (AL-35-2)

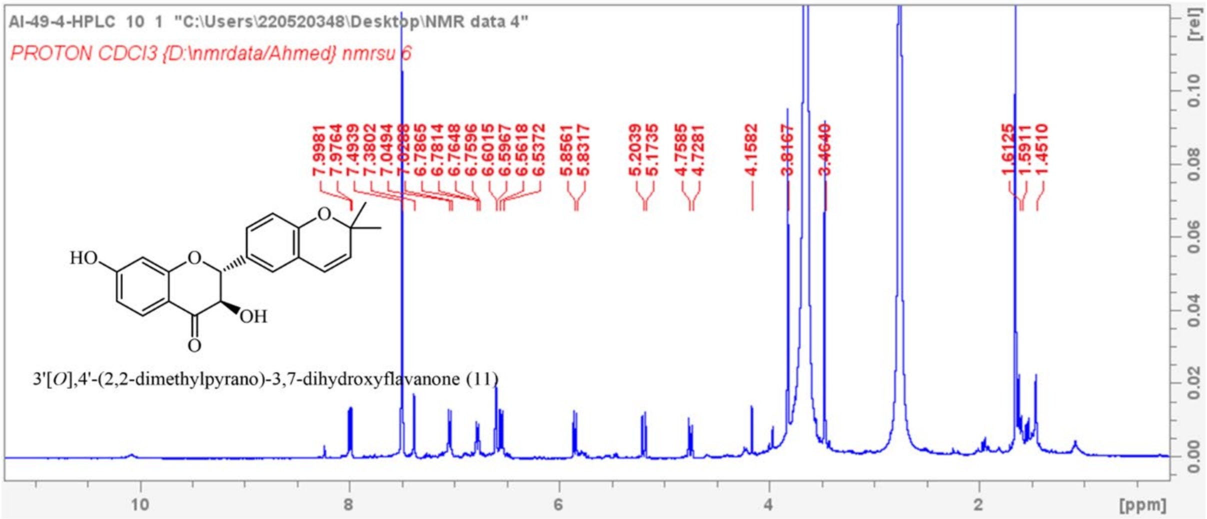

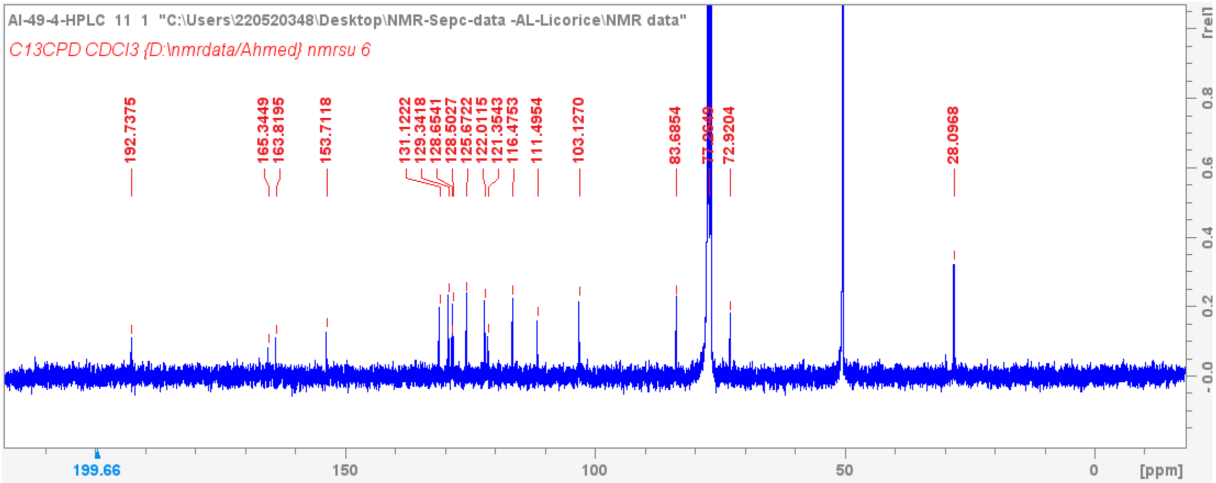

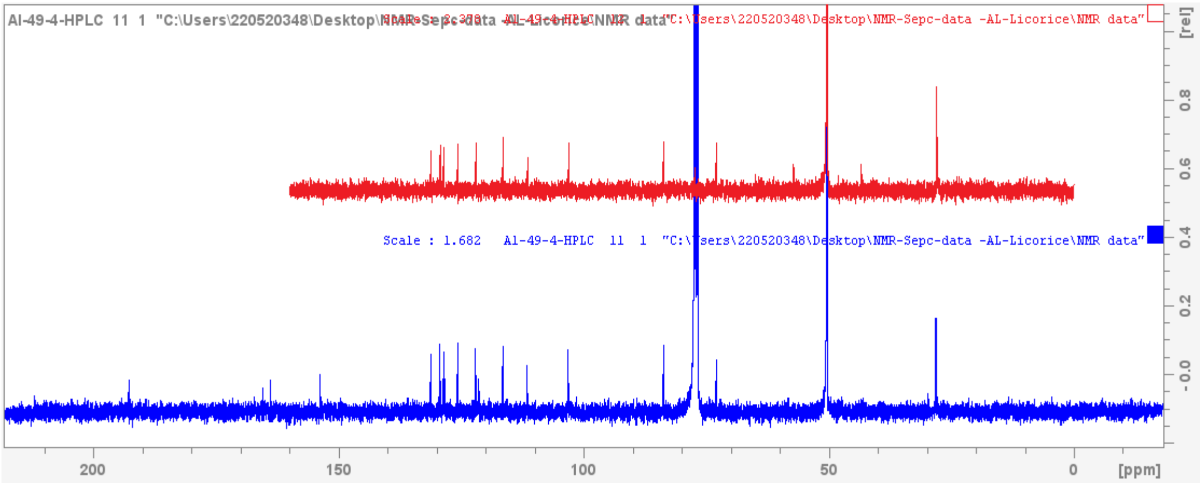

Figure S1.11; ^{1}H and ^{13}C-NMR (400 MHz, CDCl3-d_6) spectrum of compound **11**; (Al-49-4)

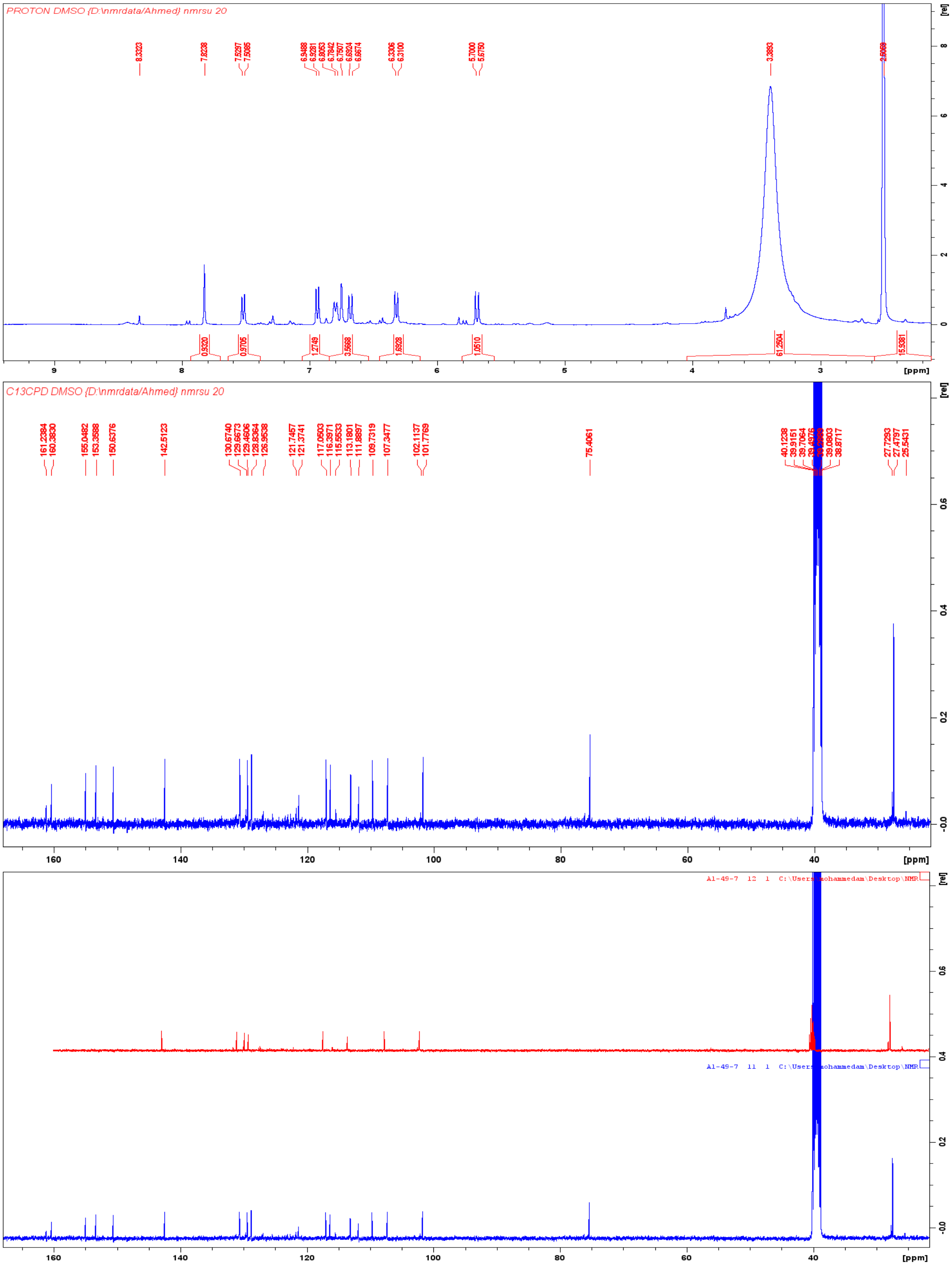

Figure S1.12; ^{1}H and ^{13}C-NMR (400 MH$_Z$, CDCl3-d_6) spectrum of compound **12**; (AL-49-7)

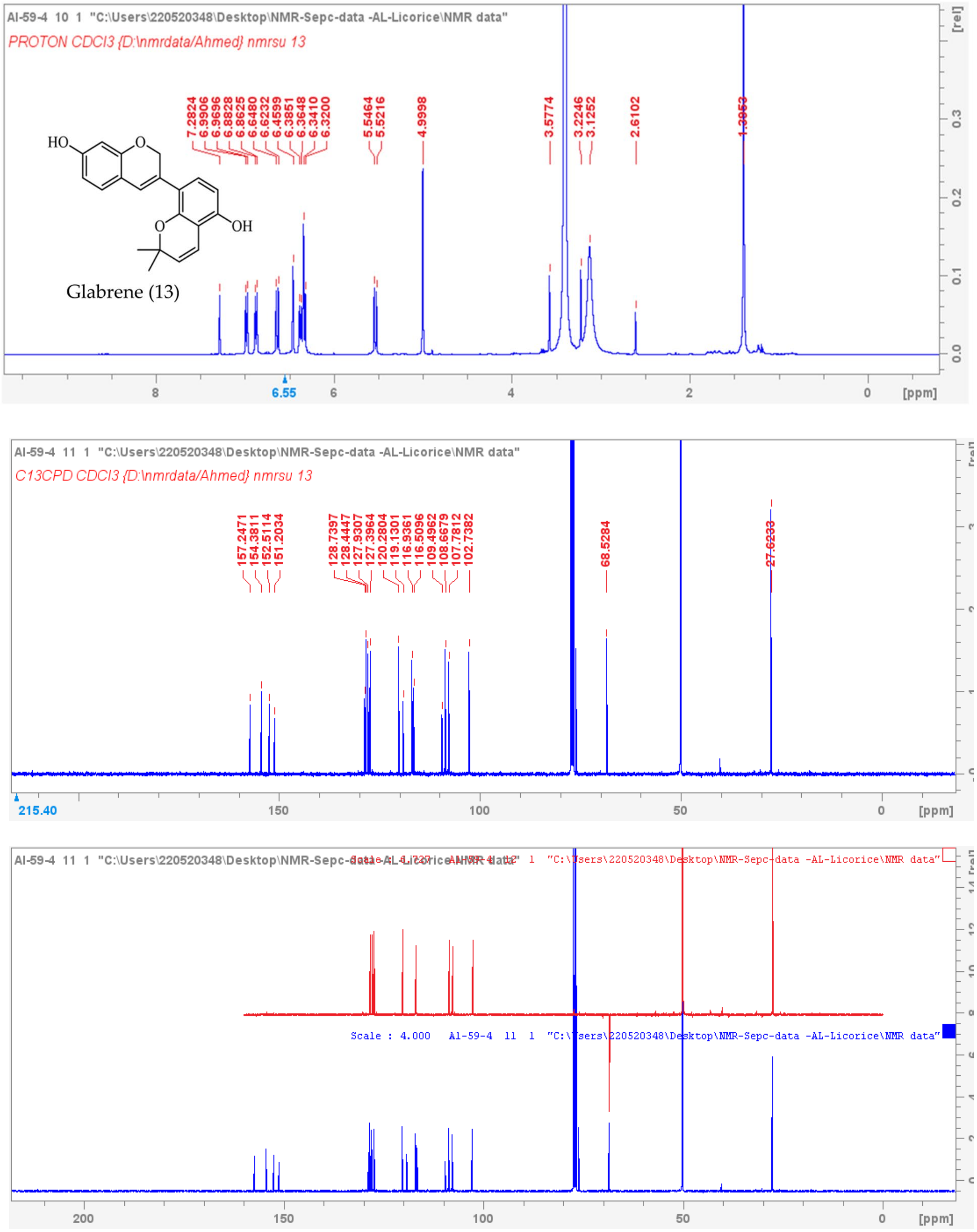

Figure S1. 13; ^{1}H and ^{13}C-NMR (400 MH$_{Z}$, CDCl3-d_6) spectrum of compound **13**; (AL-59-4)

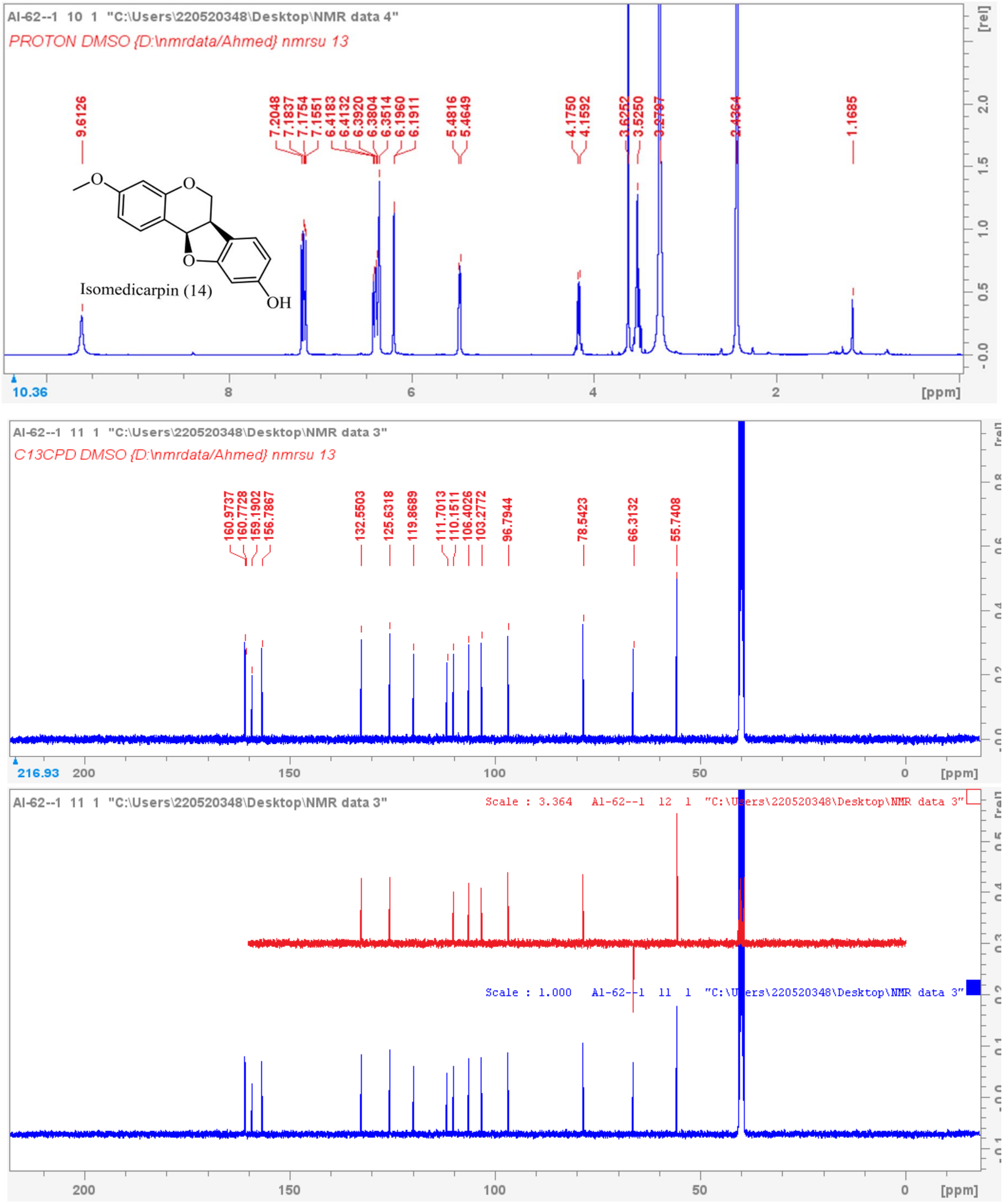

Figure S1. 14; ^{1}H and ^{13}C-NMR (400 MH$_Z$, DMSO-d_6) spectrum of compound **14**; (AL-62--1)

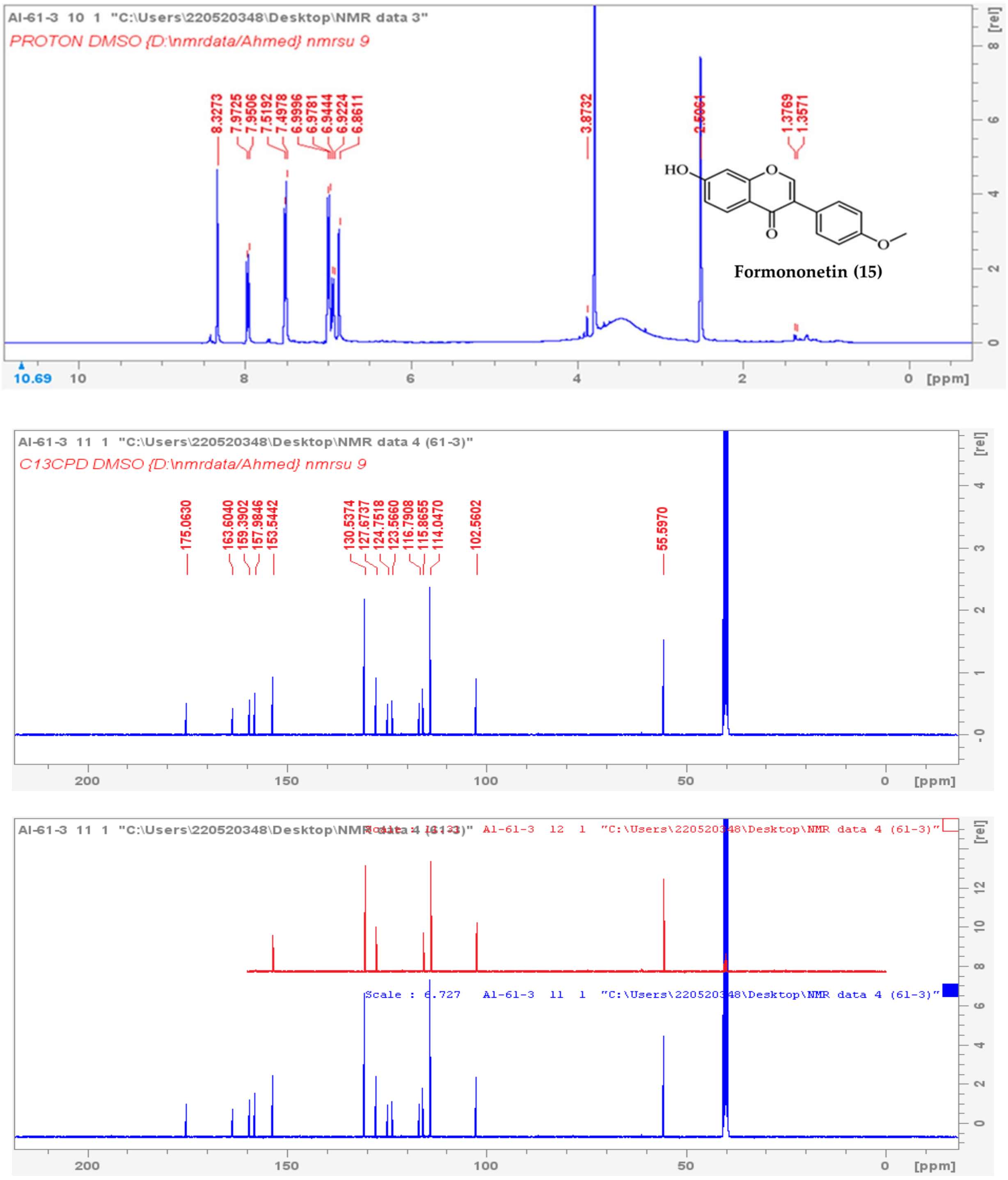

Figure S1. 15; ^{1}H and ^{13}C-NMR (400 MHz, DMSO-d_6) spectrum of compound **15**; (AL-61-3)

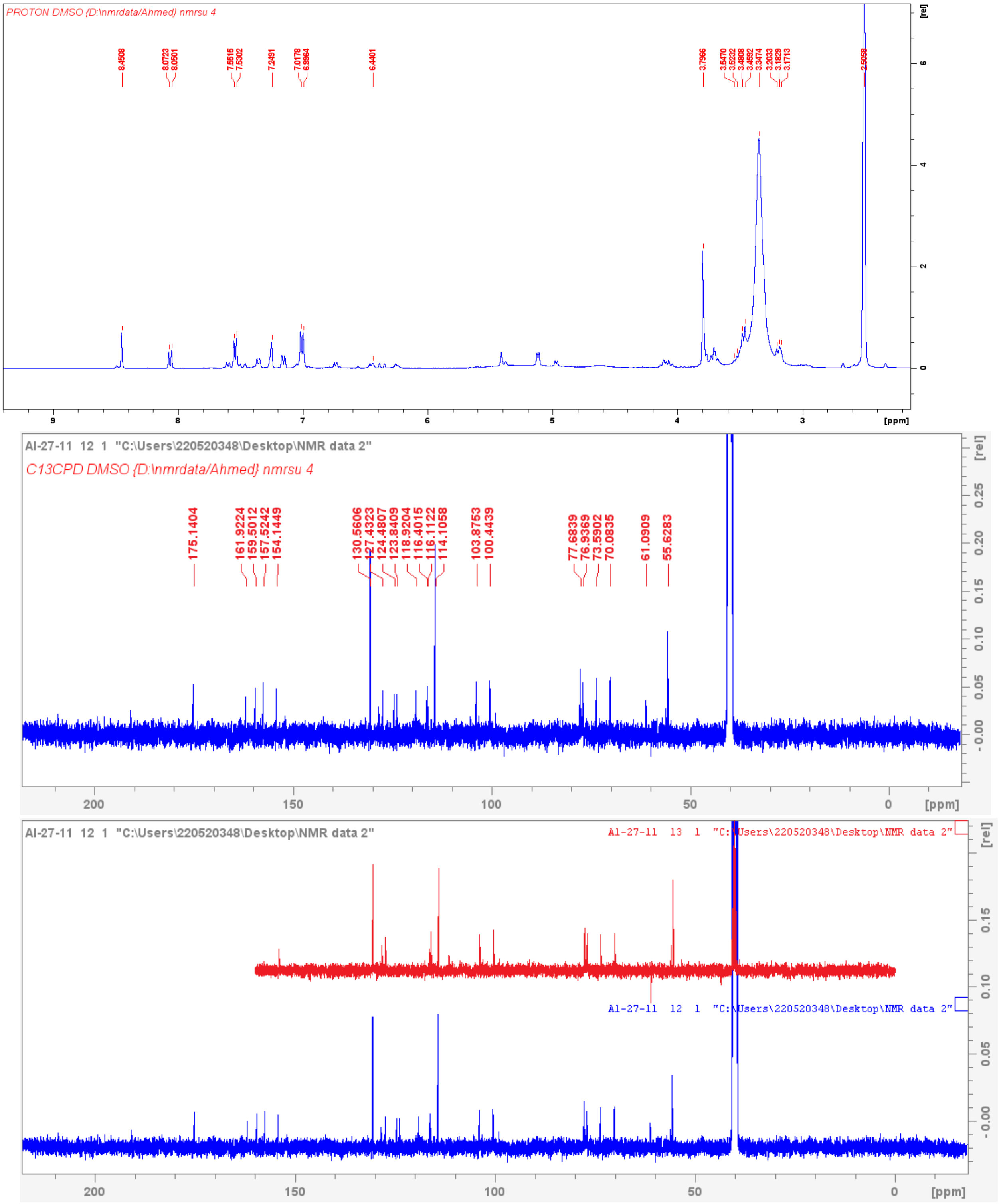

Figure S1. 16; ^{1}H and ^{13}C-NMR (400 MHz, DMSO-d_6) spectrum of compound **16**; (AL-27-11)

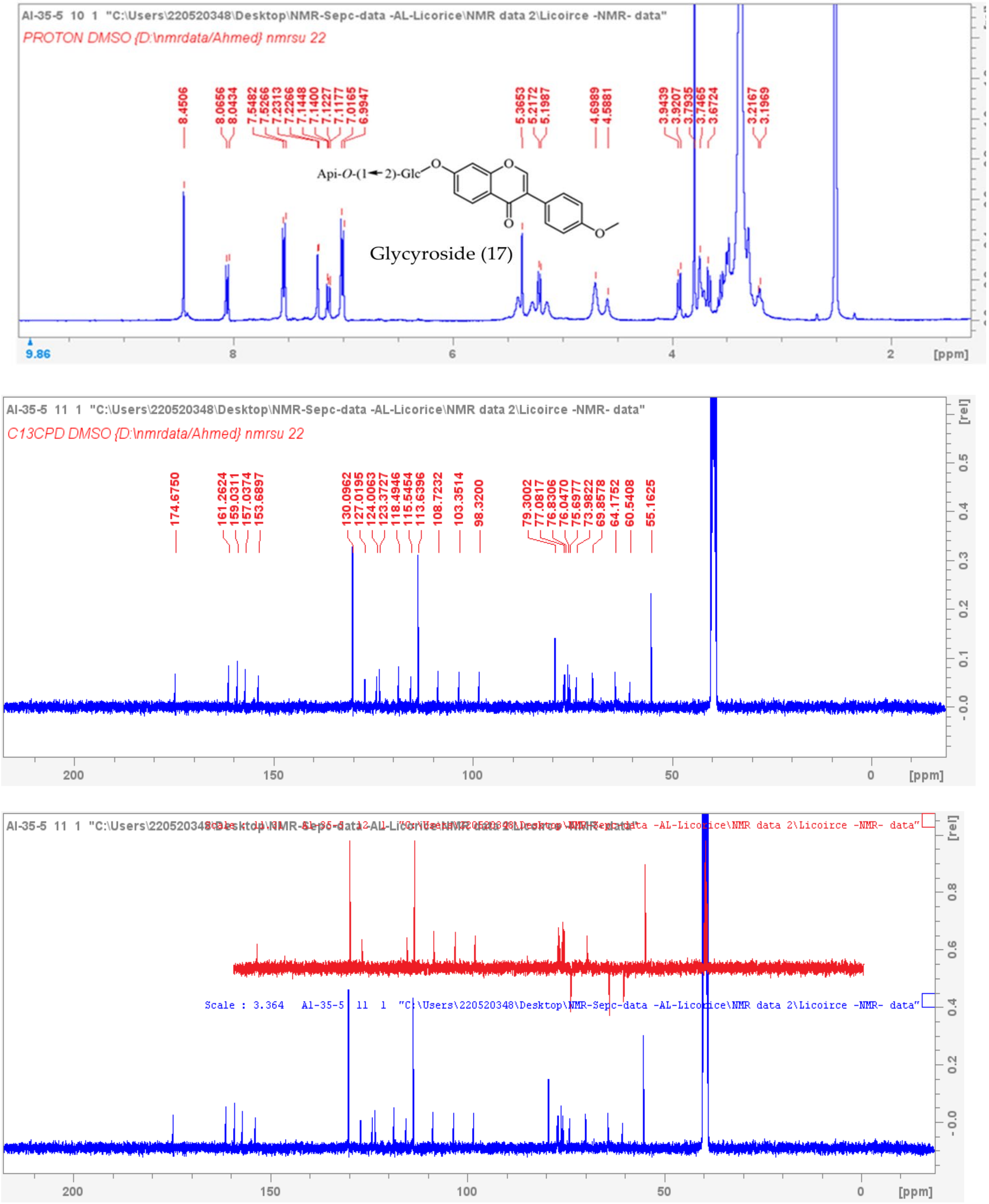

Figure S1. 17; ^{1}H and ^{13}C-NMR (400 MHz, DMSO-d_6) spectrum of compound **17**; (AL-35-5)

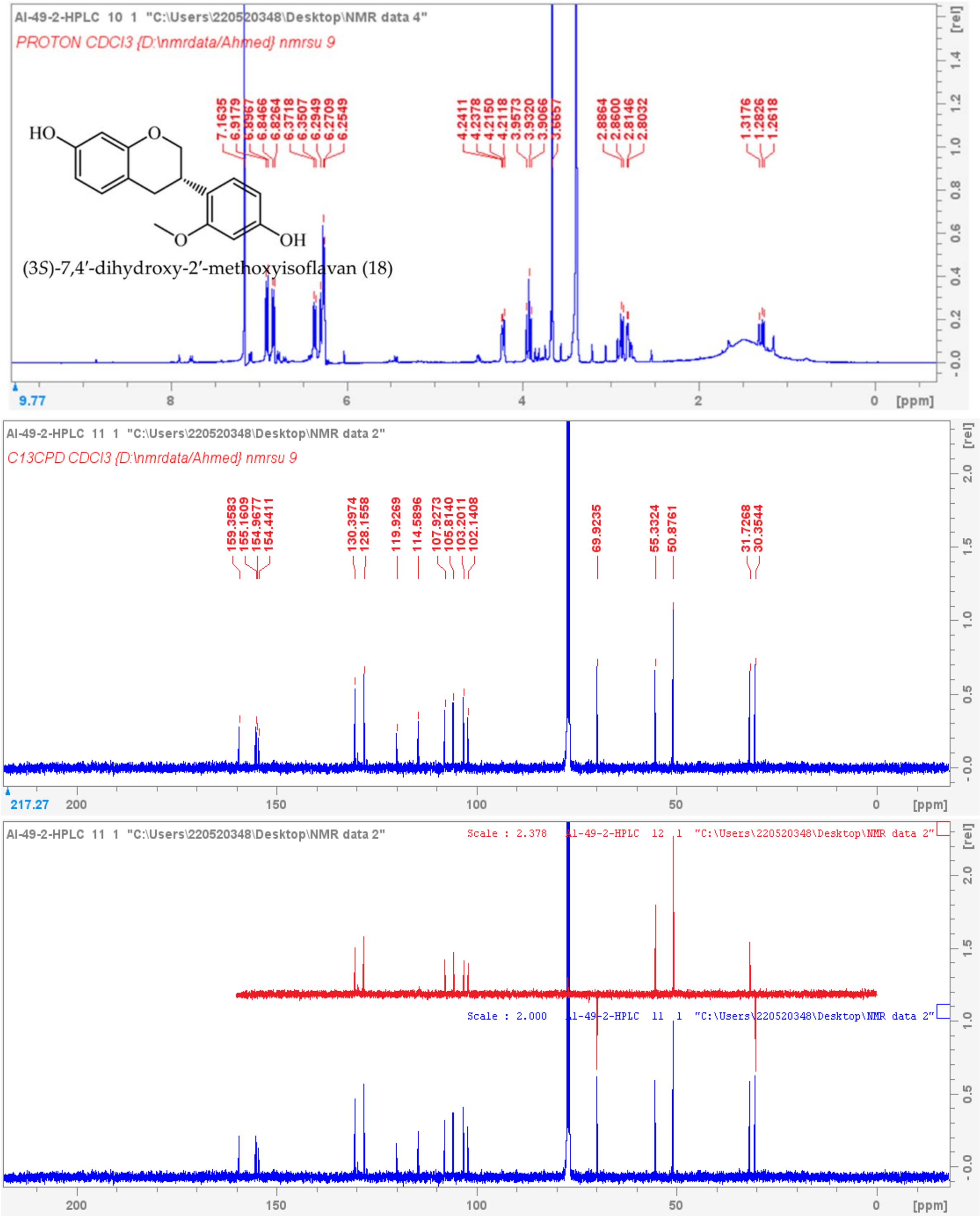

Figure S1. 18; ^{1}H and ^{13}C-NMR (400 MHz, CDCl3-d_6) spectrum of compound **18**; (AL-49-2)

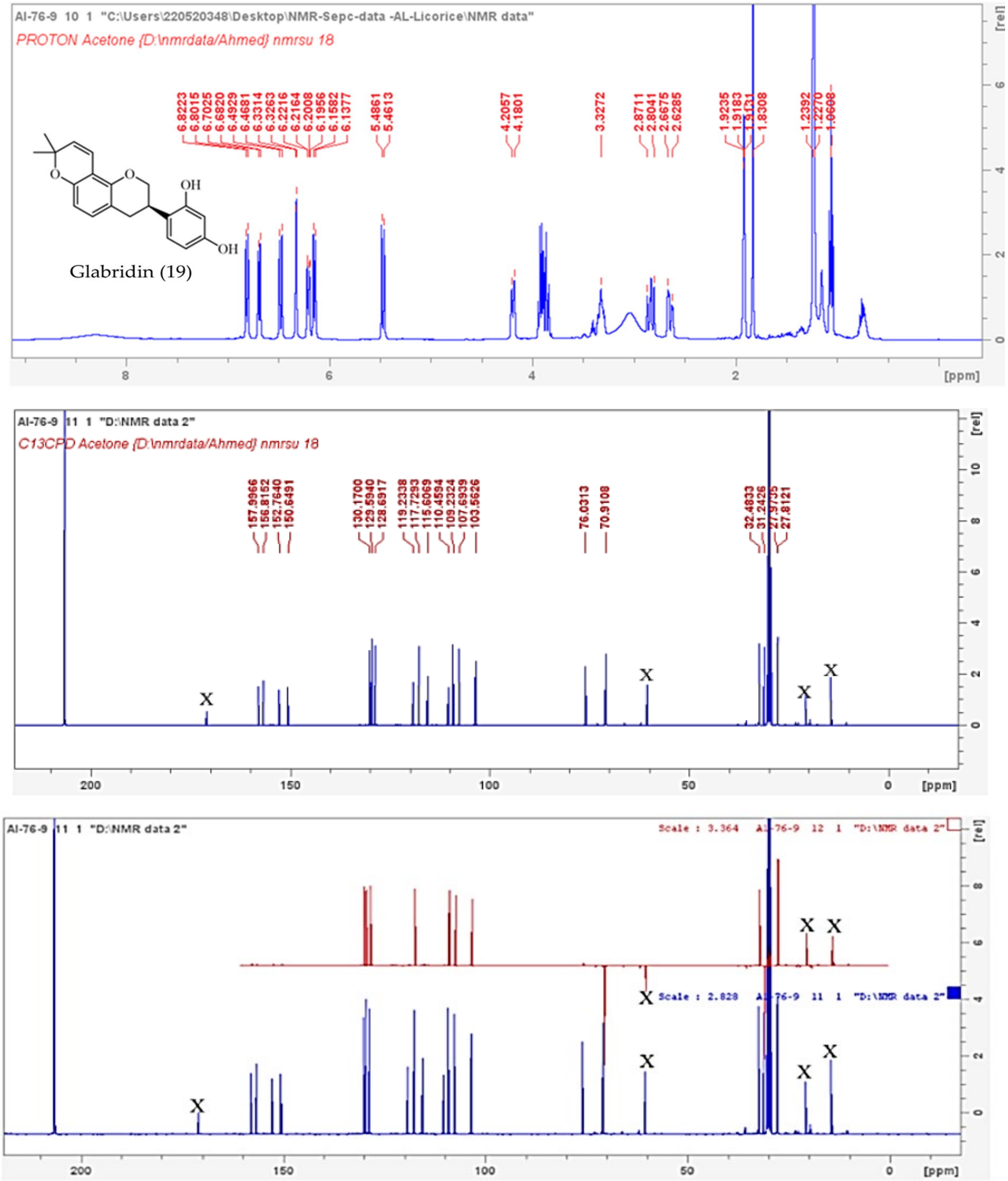

Figure S1. 19; ^{1}H and ^{13}C-NMR (400 MHz, Acetone-d_6) spectrum of compound **19**; (AL-76-9)

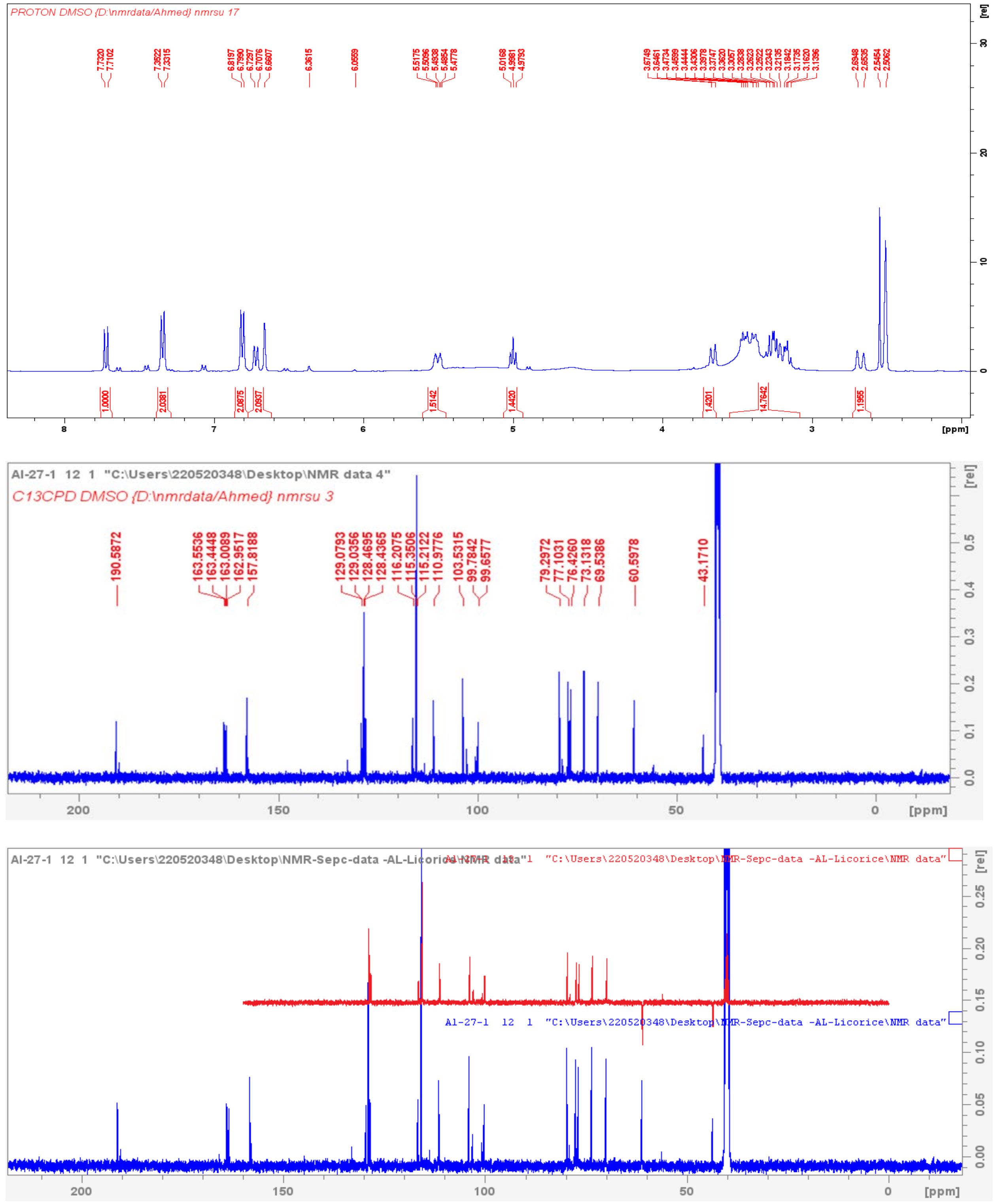

Figure S1. 20; ^{1}H and ^{13}C-NMR (400 MHz, DMSO-d_6) spectrum of compound **20**; (AL-27-1)

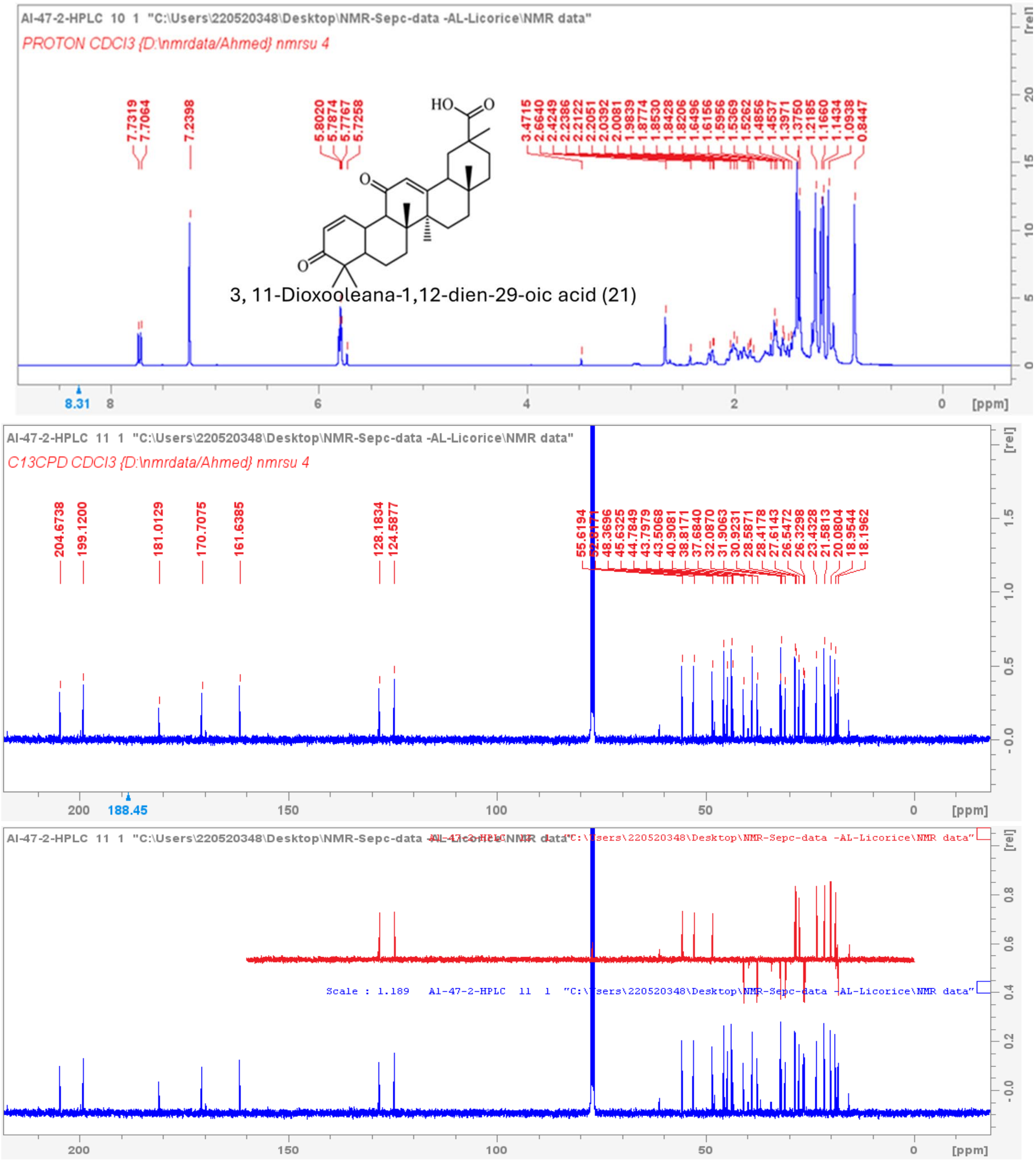

Figure S1. 21; ^{1}H and ^{13}C-NMR (400 MH$_{Z}$, CDCl3-d_6) spectrum of compound **21**; (AL-47-2)

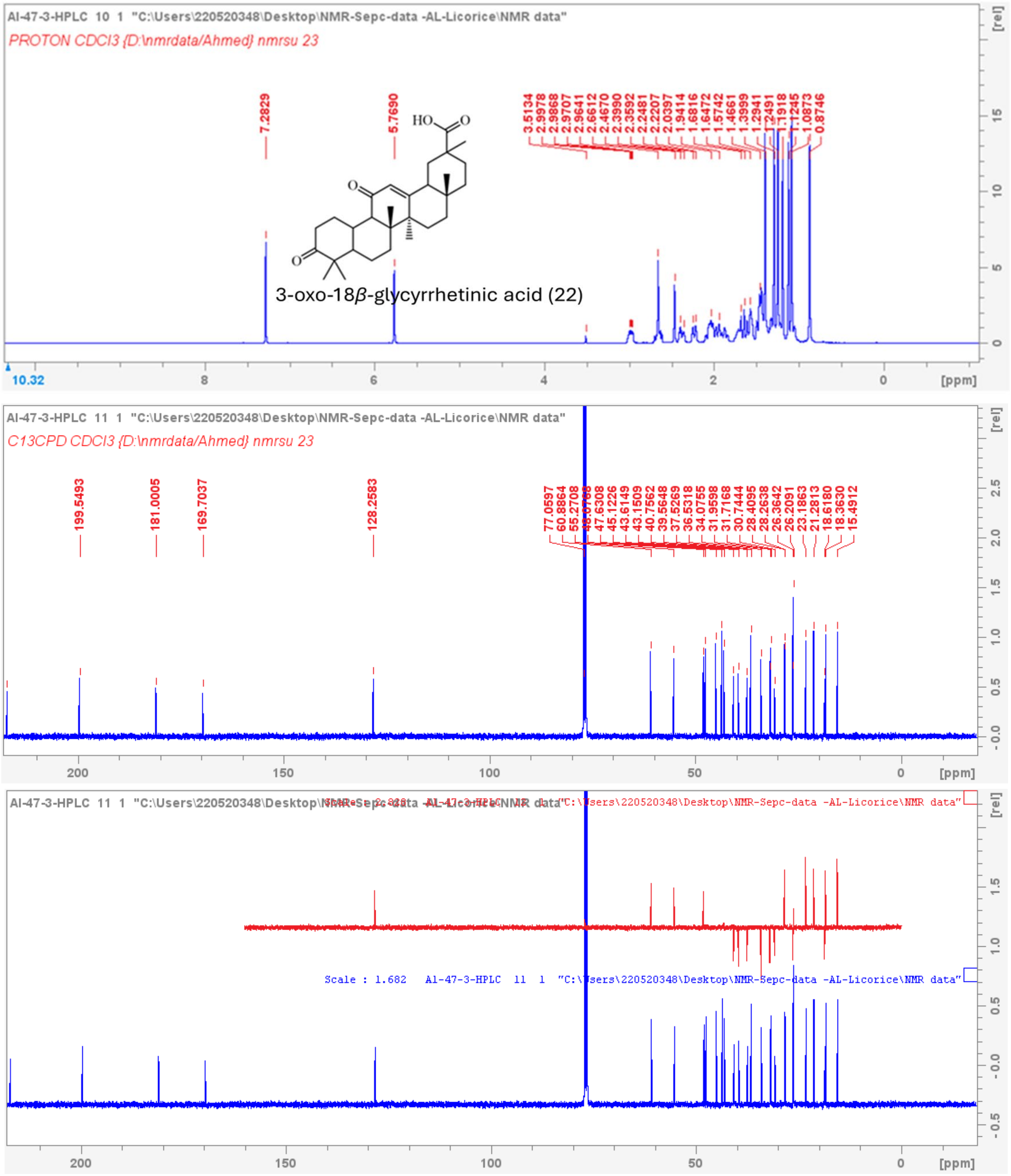

Figure S1. 22; ^{1}H and ^{13}C-NMR (400 MHz, CDCl3-d_6) spectrum of compound **22**; (AL-47-3)

Scheme S1. The details of the isolation processes for compounds 1−22 from licorice.

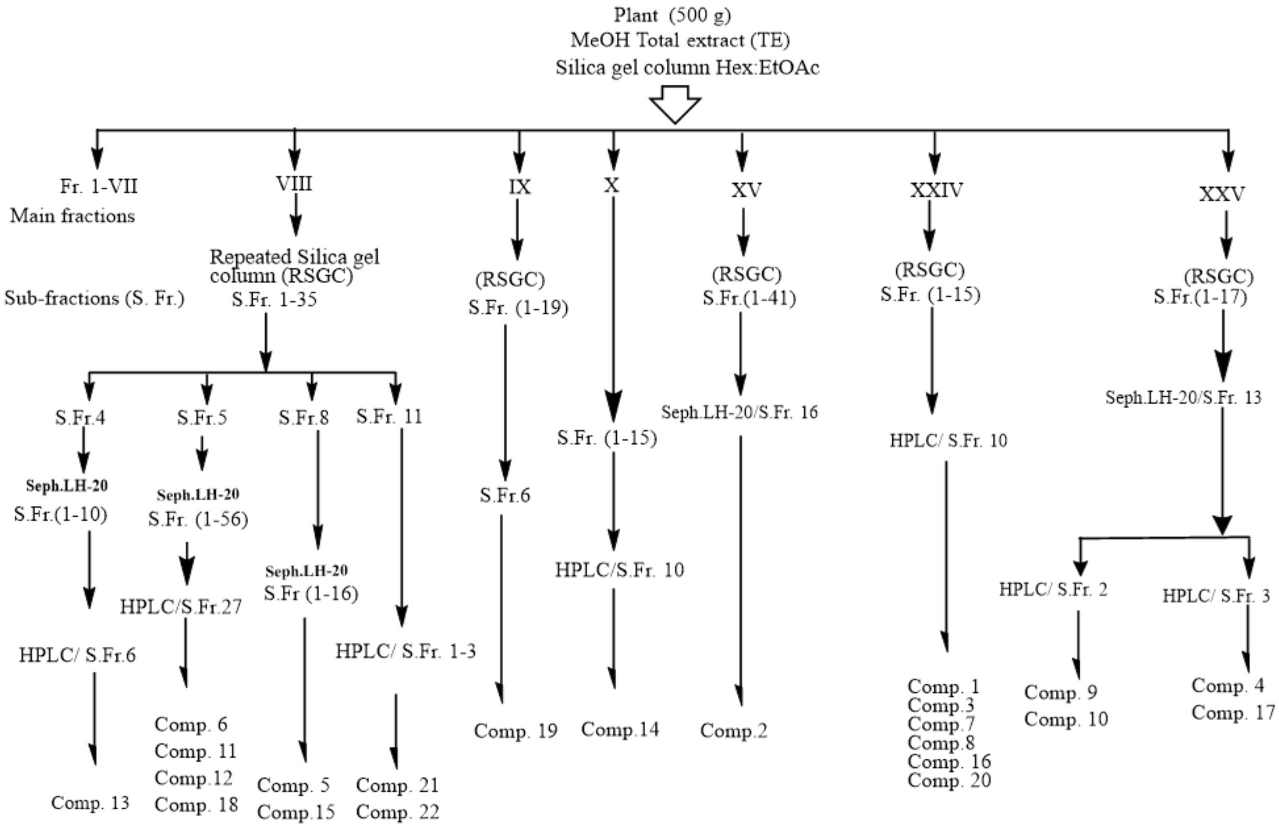

Supplement: Supplementary file 1 [file pharmaceuticals-17-00852-s001.zip › pharmaceuticals-3021032-supplementary.pdf]
